# Characterization of the AP2/ERF Transcription Factor Family and Expression Profiling of DREB Subfamily under Cold and Osmotic Stresses in *Ammopiptanthus nanus*

**DOI:** 10.3390/plants9040455

**Published:** 2020-04-04

**Authors:** Shilin Cao, Ying Wang, Xuting Li, Fei Gao, Jinchao Feng, Yijun Zhou

**Affiliations:** College of Life and Environmental Sciences, Minzu University of China, Beijing 100081, China; 18301207@muc.edu.cn (S.C.); wyxm12138@163.com (Y.W.); 15210636339@163.com (X.L.); jchfeng@263.net (J.F.)

**Keywords:** Ammopiptanthus nanus, AP2/ERF, transcription factor, gene family, osmotic stress, cold stress

## Abstract

APETALA2/ethylene-responsive factor (AP2/ERF) is one of the largest transcription factor (TF) families in plants, which play important roles in regulating plant growth, development, and response to environmental stresses. *Ammopiptanthus nanus*, an unusual evergreen broad-leaved shrub in the arid region in the northern temperate zone, demonstrates a strong tolerance to low temperature and drought stresses, and AP2/ERF transcription factors may contribute to the stress tolerance of *A. nanus*. In the current study, 174 AP2/ERF family members were identified from the *A. nanus* genome, and they were divided into five subfamilies, including 92 ERF members, 55 dehydration-responsive element binding (DREB) members, 24 AP2 members, 2 RAV members, and 1 Soloist member. Compared with the other leguminous plants, *A. nanus* has more members of the DREB subfamily and the B1 group of the ERF subfamily, and gene expansion in the AP2/ERF family is primarily driven by tandem and segmental duplications. Promoter analysis showed that many stress-related *cis*-acting elements existed in promoter regions of the DREB genes, implying that MYB, ICE1, and WRKY transcription factors regulate the expression of DREB genes in *A. nanus*. Expression profiling revealed that the majority of DREB members were responsive to osmotic and cold stresses, and several DREB genes such as *EVM0023336.1* and *EVM0013392.1* were highly induced by cold stress, which may play important roles in cold response in *A. nanus*. This study provided important data for understanding the evolution and functions of AP2/ERF and DREB transcription factors in *A. nanus*.

## 1. Introduction

Plants are inevitably subjected to various adverse conditions in their life cycle, such as cold stress, drought stress, and salt stress, which negatively affect their growth, development, and yield [1]. Plants respond to environmental stresses at both cellular and molecular levels by regulating the expression of many genes via a variety of signal transduction pathways, and several transcription factor (TF) families, including APETALA2/ethylene-responsive factor (AP2/ERF), WRKY, NAC (NAM, ATAF1, 2, and CUC2), and MYB (myeloblastosis oncogene), play crucial roles in stress signal transduction [2].

AP2/ERF family is one of the largest families in the plant kingdom, and proteins in this family contain at least one AP2/ERF DNA-binding domain that consists of 60~70 conserved amino acids [3]. The AP2 domain is important for the binding activity to the *cis*-acting elements including the dehydration-responsive element/C-repeat (DRE/CRT) and the GCC box in the promoter regions of target genes of AP2/ERF TFs [4,5]. The AP2/ERF family is divided into five subfamilies, i.e., ethylene-responsive element binding factors (ERFs), dehydration-responsive element binding (DREB), AP2, related to ABI3/VP1 (RAV), and Soloist, based on the characteristics of their conserved domains. Both the ERF and DREB subfamily members contain a single AP2 domain, but important differences exist in some specific amino acid sites of their AP2 domains [6]. The AP2 subfamily members contain two adjacent AP2 domains, and the RAV subfamily members possess both an AP2 domain and a B3 domain. The Soloist subfamily TFs also contain an AP2 domain, but their AP2 domains exhibit low homology with other AP2/ERF members [7]. AP2/ERF TFs have been shown to play essential roles in plant growth and development [8,9,10] and abiotic stress responses [2,11]. Genome-wide identification and analysis of the AP2/ERF family have been conducted in several plant species including *Arabidopsis thaliana* and rice [7], *Vitis vinifera* [12], *Glycine max* [13], *Medicago truncatula* [14], *Eucalyptus grandis* [15], poplar [16], cucumber [17], *Ricinus communis* [18], *Brassica rapa* [19], *Setaria italica* [20], *Phaseolus vulgaris* [21], and *Salix arbutifolia* [22], and these works have greatly promoted an understanding of the biological functions of AP2/ERF family TFs.

Many members of the AP2/ERF family, especially the DREB subfamily, have been shown to take part in abiotic stress responses [23,24]. The DREB subfamily, also called the C-repeat binding factor (CBF) family, is composed of six groups (A1 to A6) based on their amino acid sequences. DREB subfamily TFs have unique conserved regions that bind to the DRE/CRT *cis*-acting elements (core nucleotide sequence: CCGAC) containing genes. Different groups of DREB subfamily TFs may regulate distinct but overlapped sets of downstream genes. In *Arabidopsis*, DREB A1 group genes were upregulated in response to low-temperature treatment [25], while members of the DREB A2 group such as DREB2A and DREB2B were induced by dehydration, high salinity, and heat shock, but not by cold stress [26]. However, with the expansion of the list of DREB subfamily TFs identified in different plant species, it is found that some DREB subfamily TFs can also be induced by a variety of stresses [27]. DREB subfamily members regulated one of the best documented cold signaling pathways by controlling the expression of a large number of cold-response related genes, including *COR* (cold regulated), *KIN* (cold induced), *RD* (responsive to desiccation), and *LEA* (late embryogenesis abundant) genes. The cold induction of DREB1s is mediated by their upstreaming transcription factors, inducer of CBF expression 1s (ICE1s), which is stabilized by cold treatment. ICE1s bind to the MYC-recognition sequences (E-box, CANNTG) of DREB1s’ promoter and activate their expression [28]. DREB subfamily genes have been intensively investigated as candidate genes in plant genetic engineering for improving tolerance to abiotic stress, and overexpression of some DREB genes in *Arabidopsis* and crop plants has resulted in increased tolerance to cold, drought, and high salinity stresses [29].

*Ammopiptanthus nanus* is a desert shrub belonging to *Ammopiptanthus*, Leguminosae. As a tertiary relict in Central Asia, *A. nanus* has developed strong tolerance to low- and high-temperature stress, water shortage, strong wind, and strong irradiation. Specifically, *A. nanus* is a rare evergreen broad-leaved shrub in the arid region in Central Asia, attracting many research interests in recent years [30,31]. With the rapid development of high-throughput sequencing technology, the high-quality genome of *A. nanus* obtained using the long-read sequencing technology was released [32], which facilitates the investigation of the molecular mechanism underlying the stress tolerance of *A. nanus*. Considering the roles of the AP2/ERF family, especially the DREB subfamily, in regulating the response to environmental stress in plants, we conjectured that some members of the DREB subfamily TFs might contribute to the strong abiotic stress tolerance in *A. nanus.* In the present study, we performed a comprehensive analysis of the AP2/ERF superfamily and expression profiling of the DREB subfamily genes under osmotic and cold stress in *A. nanus*. The results will be helpful for understanding the biological functions of AP2/ERF family TFs and DREB subfamily TFs.

## 2. Results

### 2.1. Identification and Phylogenetic Analysis of AP2/ERF Family Genes in A. nanus 

**Figure 1:**
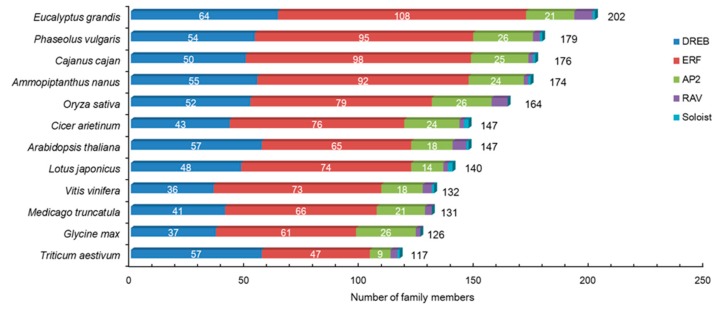
The number of APETALA2/ethylene-responsive factor (AP2/ERF) family members in *Ammopiptanthus nanus* and some other plant species.

A total of 174 putative full-length AP2/ERF family genes (AnAP2/ERF) were identified in *A. nanus* (Table S1). The total number of AP2/ERF family genes in *A. nanus* is comparable to those of *P. vulgaris* (179 members) [21] and *Cajanus cajan* (176 members) [33], two leguminous plants, but is higher than those of *A. thaliana* (147 members) [13], *Oryza sativa* (164 members) [13], *V. vinifera* (132 members) [14], and several leguminous plants including *G. max* (126 members) [15], *Triticum aestivum* (117 members) [34], *Cicer arietinum* (147 members) [33], and *M. truncatula* (131 members) [14], and is less than that of *E. grandis* (202 members) [15] (Figure 1). 

The 174 putative AnAP2/ERF genes encode proteins ranging from 135 to 705 aa in length. The amino acid length of most DREB and ERF subfamily members are between 135 and 360, while those of all AP2 and RAV subfamily members are longer than 360. The pI values of the predicted AP2/ERF TFs range from 4.39 to 10.13, with 114 (65.52%) proteins with pI values > 7. 

**Figure 2:**
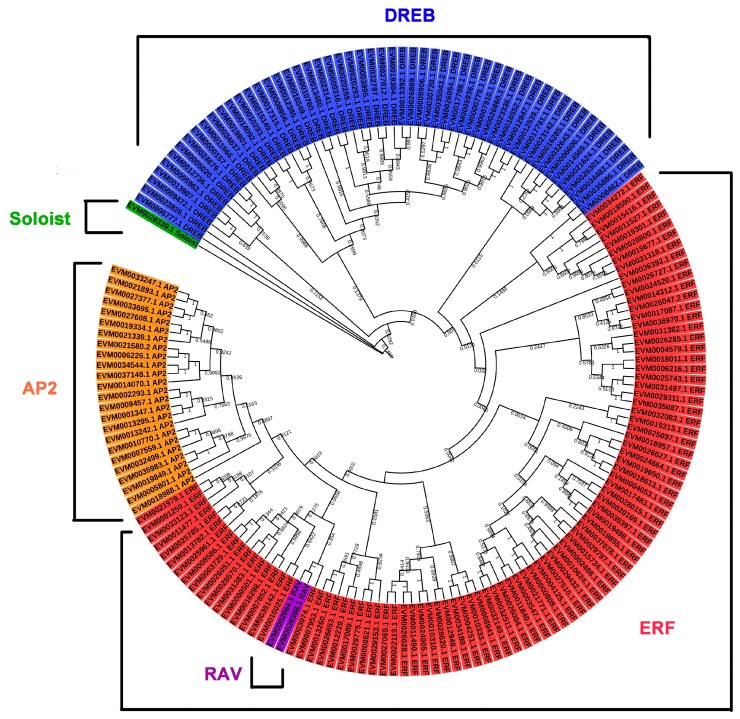
Phylogenetic analysis of AP2/ERF superfamily members in *A. nanus.*

**Figure 3:**
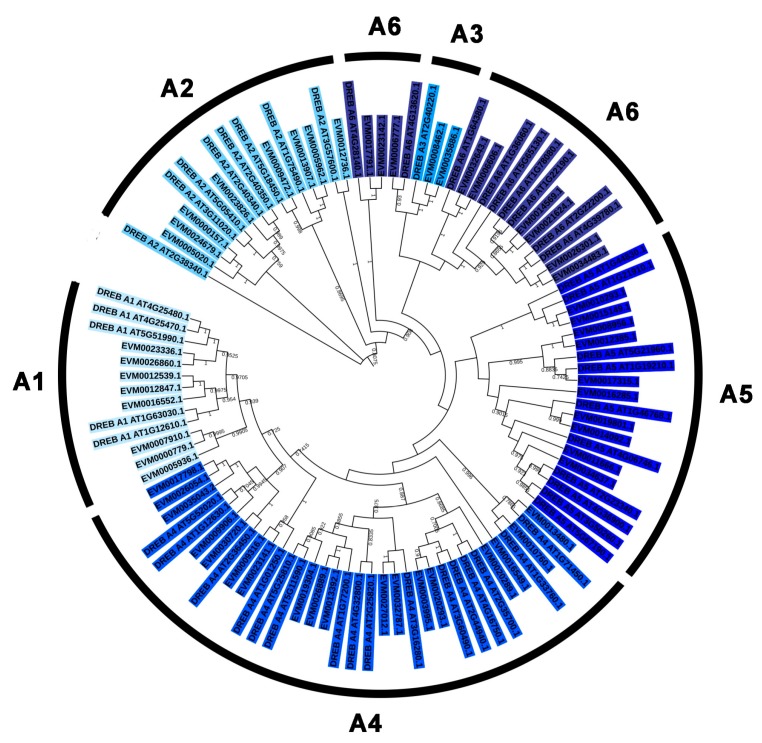
Phylogenetic analysis of dehydration-responsive element binding (DREB) subfamily members in *A. nanus* and *Arabidopsis thaliana*.

**Figure 4:**
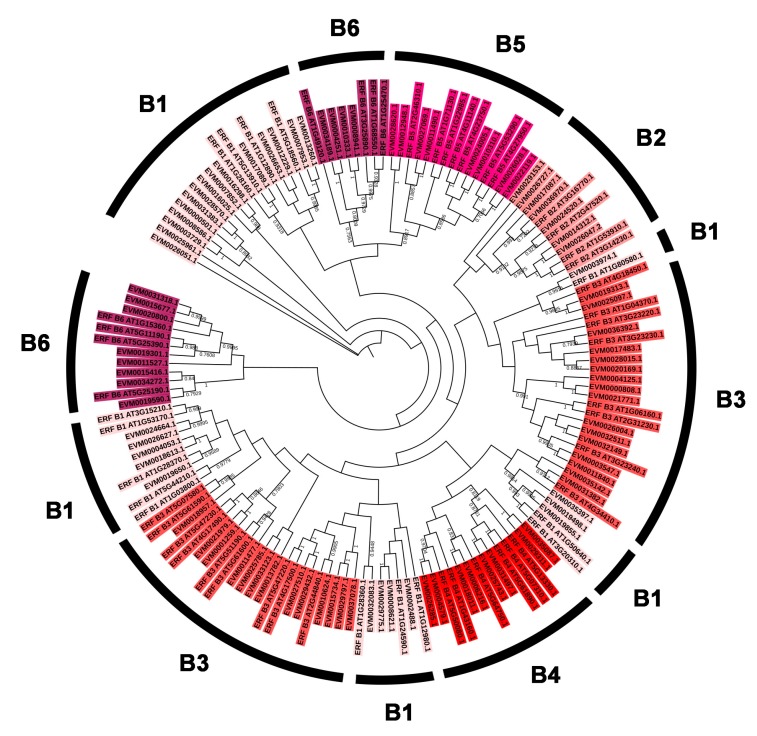
Phylogenetic analysis of ERF subfamily members in *A. nanus* and *A. thaliana*.

To classify the evolutionary relationships of the AP2/ERF family members in *A. nanus*, the full-length amino acid sequences of the putative proteins were aligned and the phylogenetic trees were constructed (Figure 2, Figure 3 and Figure 4). Based on the phylogenetic tree of the AnAP2/ERF family members, all AnAP2/ERF family members were classified into five subfamilies: 92 ERF members, 55 DREB members, 24 AP2 members, 2 RAV members, and 1 Soloist member. The DREB and ERF subfamilies were further divided into six groups (A1 to A6, B1 to B6). Groups A1, A2, A3, A4, A5, and A6 contain 8, 8, 2, 18, 10, and 9 members, respectively, and groups B1, B2, B3, B4, B5, and B6 contain 28, 7, 29, 8, 8, and 12 members, respectively [35] (Figure 3 and Figure 4). 

### 2.2. Chromosomal Locations of the AnAP2/ERF Family Genes 

**Figure 5:**
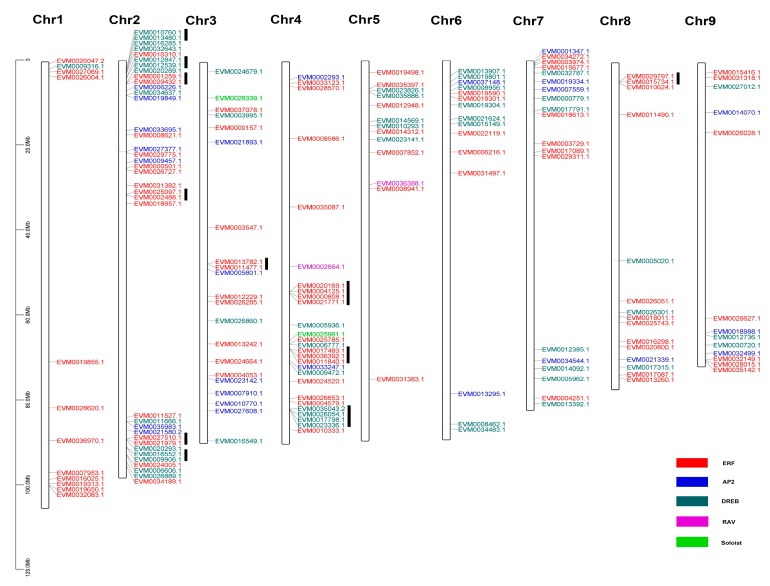
Distribution of the AP2/ERF family genes on the *A. nanus* chromosomes. The black vertical bars to the right of the gene names indicate tandem duplication.

A total of 172 AnAP2/ERF genes were located on the 9 chromosomes of *A. nanus* (Figure 5), and the precise locations of 2 AnAP2/ERF genes, *EVM0029153.1* and *EVM0032511.1*, were not determined. All 172 genes were distributed across all 9 chromosomes but not evenly. More AnAP2/ERF genes were located on chromosomes 2 and 4 (37 and 27, respectively), while only 12 AnAP2/ERF genes were distributed on chromosome 1. 

### 2.3. Gene Structure and Conserved Motifs of the AnAP2/ERF Family Transcription Factors

To analyze the conservation of the predicted AnAP2/ERF family TFs, the deduced amino acid sequences of AP2 domains were aligned with the corresponding sequences in *A. thaliana* AP2/ERF TFs, which have been identified previously [7] (Figures S1–S4). All of the deduced amino acid sequences of the AP2 domain of the DREB subfamily proteins have a WLG motif (W33, L34, G35), except for *EVM0000157.1*, which contains V34. Of the 6 groups of the DREB subfamily, A2 and A6 exhibit a higher level of conservation than other groups (Figure S1). 

The sequence alignments of the ERF B1 to B6 groups showed that most of the AP2 domains of ERF members from *A. nanus* and *A. thaliana* contain residues G3, R7, G10, E15, I16, W28, G30, T31, and Y42, and that all the AP2 domains of these ERF members contain residues W28, G30, T31, and Y42. The WLG motif (W28, L29, G30) is also present in almost all the AP2 domains of the ERF subfamily, with the exception of *EVM0025962.1*, *EVM0032511.1*, and *EVM0032149.1,* which contain YLG, WIG, and WIG, respectively. Of the 6 groups of the ERF subfamily, groups B4 and B5 exhibit a high level of conservation, while the AP2 domains of group B6 show a considerable degree of variation (Figure S2).

EVM0002664.1 and EVM0036388.1 contain the AP2/ERF domain and the B3 domain, thus they were assigned to the RAV subfamily. Twenty-four AnAP2/ERF family members contain two AP2/ERF domains, therefore they are classified into the AP2 subfamily. The AP2 domains of *A. nanus* RAV and AP2 subfamily members share many conservative amino acid residues with those of *A. thaliana*. The WLG motif in the RAV subfamily in *A. nanus* is highly conserved, while in the AP2 subfamily the motif is presented as YLG (Figures S3 and S4).

**Figure 6:**
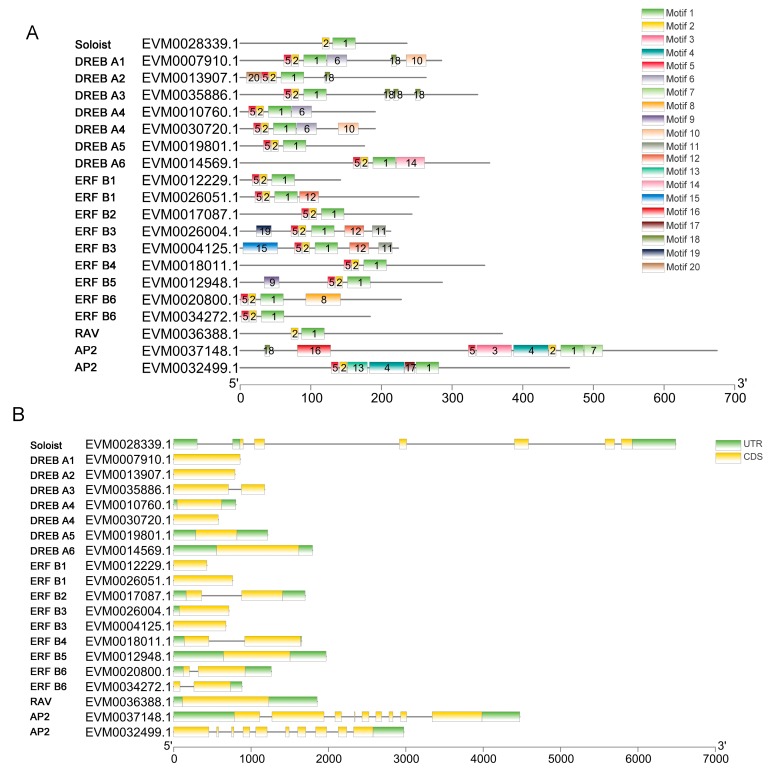
Representative motif compositions and gene structures of the AP2/ERF family members in *A. nanus*. One or two members from Soloist, RAV, and AP2 subfamilies and one member from each ERF and DREB subfamilies were used to show the typical motif compositions and gene structures of members in the subfamily or the group. (**A**) Conserved motif analysis of the AnAP2/ERF family transcription factors. All motifs were identified using the MEME database. (**B**) Gene structure of AnAP2/ERF family members. Exons and introns are represented by yellow boxes and black lines, respectively, and the untranslated region (UTR) is shown in green.

The conserved motifs in AnAP2/ERF family proteins were further investigated using MEME [36], and a total of 20 conserved motifs were found (Figure 6, Figure S5 and S6). Motifs 1, 2, and 5, namely the three motifs located in the AP2 domain regions, were found in nearly all AP2/ERF family members. In the AP2 subfamily of *A. nanus*, motifs 1~5 were mainly located in the region of two consecutive AP2 domains. It is noteworthy that the AnAP2/ERF members in the same subfamily often have the same set of motifs, and many predicted motifs were distributed in the AnAP2/ERF family in a subfamily-specific manner. For example, motif 20 was only detected in group A1 of subfamily DREB, motif 14 was only detected in group A6 of subfamily DREB, motif 6 was found in groups A1, A4, and A5 of subfamily DREB, motifs 11 and 12 were found in group B3 of subfamily ERF, and motif 9 was specifically detected in groups B5 and B6 of subfamily ERF.

Gene structural analyses revealed that most DREB and ERF subfamily members and all members in the RAV subfamily were intronless (Figure 6 and Figure S6). Of the 55 members in the DREB subfamily, 53 contained one or no introns (41 members contained no introns); one member contained two introns; and one member contained six introns, which belonged to group A2 of subfamily DREB. In the ERF subfamily, 87 members contained one or no introns (56 members contained no introns), the rest of the 5 members contained 2~4 introns. In contrast, the AP2 and Soloist subfamilies contained more introns than the DREB, ERF, and RAV subfamilies. All the AP2 members contained at least 7 introns and at most 9, and only one member of the Soloist subfamily contained 5 introns.

### 2.4. Duplication and Divergence of AP2/ERF Family Gene in *A. nanus*

Tandem and segmental duplication play important roles in the evolution of large gene families in plants. To investigate the evolution of the AnAP2/ERF gene family, tandem duplication events in the AnAP2/ERF gene family were analyzed. We found 27 AP2/ERF family genes present in tandem duplication in the *A. nanus* genome, including 17 ERF subfamily genes (17/92, 18.48%) and 10 DREB subfamily genes (10/55, 18.18%) (Table S2). These AnAP2/ERF genes were located in 11 tandem duplication clusters on 4 chromosomes, i.e., chromosomes 2, 3, 4, and 8 (Figure 5). Most tandem duplication gene clusters were distributed on chromosomes 2 and 4; specifically, there were 12 AP2/ERF family genes in the 3 tandem duplication gene clusters on chromosome 4 of *A. nanus*. It is noteworthy that all the genes with tandem duplication relationships were members of groups B1 and B3 in subfamily ERF and groups A1 and A4 in subfamily DREB.

**Figure 7:**
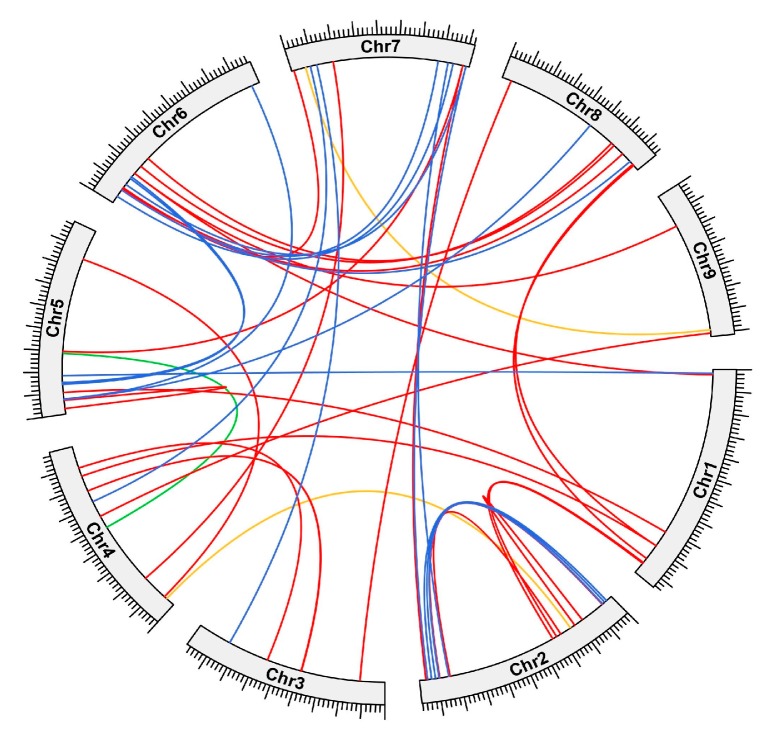
Distribution of segmentally duplicated AnAP2/ERF genes on *A. nanus* chromosomes. Paralogous pairs in the DREB subfamily are shown in blue, those of the ERF subfamily are shown in red, those of the AP2 subfamily are shown in orange, and those of the RAV subfamily are shown in green.

We also investigated the segmental duplication of the AP2/ERF gene family in the *A. nanus* genome using synteny analysis (Figure 7). A total of 83 AP2/ERF genes were found to be present in tandem duplication in the *A. nanus* genome, including 30 DREB subfamily genes (30/55, 54.55%), 47 ERF subfamily genes (47/92, 51.09%), 4 AP2 subfamily genes (4/24, 16.67%), and RAV subfamily genes (2/2, 100%) (Table S3).

**Figure 8:**
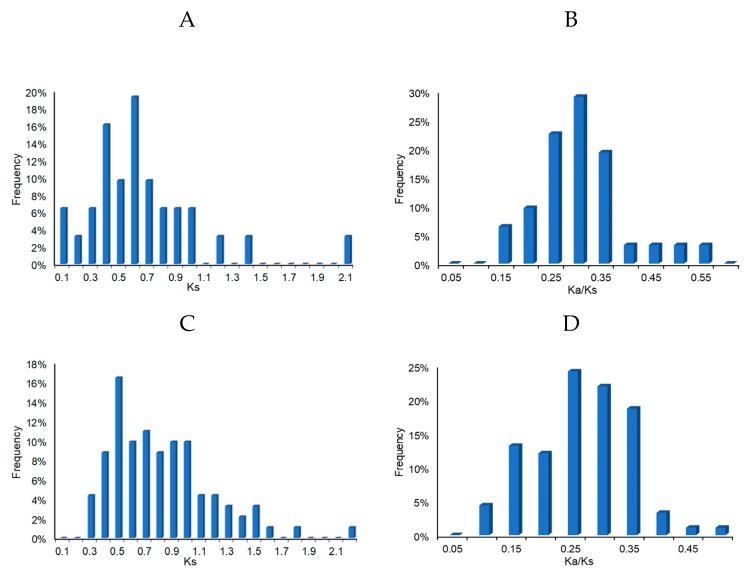
Ks and Ka/Ks value distributions of the AP2/ERF family genes in the genomes of *A. nanus* and *Glycine max*. Distribution of Ks and Ka/Ks values were calculated from the paralogous gene pairs in the *A. nanus* genome (**A** and **B**), and the orthologous gene pairs between the *A. nanus* and *G. max* genomes (**C** and **D**). Four orthologous gene pairs with Ks > 2.5 were not used for preparing the above figure.

The rate of non-synonymous (Ka) versus synonymous (Ks) is a common index to judge the existence of selection pressure. Ka/Ks = 1 stands for neutral selection, Ka/Ks >1 indicates accelerated evolution with positive selection, and Ka/Ks <1 means purifying selection [37]. Using the reciprocal best BLAST algorithm, a total of 32 paralogous pairs in AnAP2/ERF family were determined and Ka, Ks, and Ka/Ks values were calculated (Table S4). The Ks distribution peak fell in the 0.50-0.60 range in *A. nanus*, and the peak of Ka/Ks values fell in the 0.25-0.30 range (Figure 8). A total of 95 orthologous pairs in the AP2/ERF gene family between *A. nanus* and *G. max* were identified (Table S5) and the peak of Ks distribution curve was in the 0.4–0.5 range. The peak of Ka/Ks values of the AP2/ERF orthologous pairs between *A. nanus* and *G. max* fell in the 0.20–0.25 range, suggesting a purifying selection between the *A. nanus* and *G. max* genomes.

### 2.5. Analysis of Putative Cis-acting Elements in Promoters of DREB Subfamily Genes 

**Figure 9:**
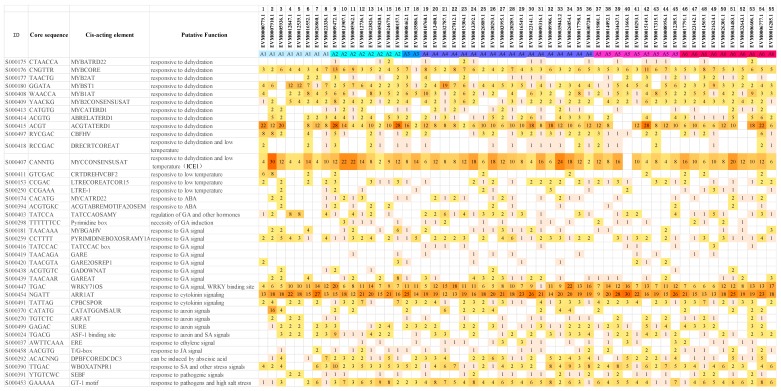
*Cis*-acting element prediction in the promoter region of the DREB subfamily genes in *A. nanus*. The color of the square depicts the quantity of the predicted *cis*-acting elements in the promoter region.

DREB subfamily TFs are believed to play important roles in plant tolerance to cold and water stresses. *Cis*-acting elements play key roles in regulating gene expression via interacting with the trans-acting element. To understand the functions and regulation network of the DREB subfamily TFs, genomic DNA 2 kb upstream of DREB subfamily genes were used to predict the putative stress-response related *cis*-acting elements (Figure 9) [38,39]. A number of *cis*-acting elements that were involved in stress response and hormone response were found, including 12 dehydration-responsive *cis*-elements, 5 low-temperature stress-responsive *cis*-elements, and 22 hormone-responsive *cis*-acting elements. The *cis*-acting elements with many copies in promoters of all DREB subfamily genes included ARR1AT (S000454), MYC/ICE1 binding site (S000407), ACGTATERD1 (S000415), and WRKY710S (S000447), which were demonstrated to participate in the response to cytokinin signal, cold, drought, and gibberellic acid (GA) signal, respectively. The large number of MYC/ICE1 binding sites and WRKY710S predicted in promoters of the DREB subfamily genes indicated that DREB genes were probably regulated under the stress environment by ICE1 and WRKY family TFs. In brief, the large amount of stress-related *cis*-acting elements found in promoters of DREB subfamily genes supported their potential biological functions in regulating low-temperature stress and drought stress response in *A. nanus*.

### 2.6. Expression Profiles of the DREB Subfamily Genes under Osmotic and Cold Stresses in *A. nanus*

**Figure 10:**
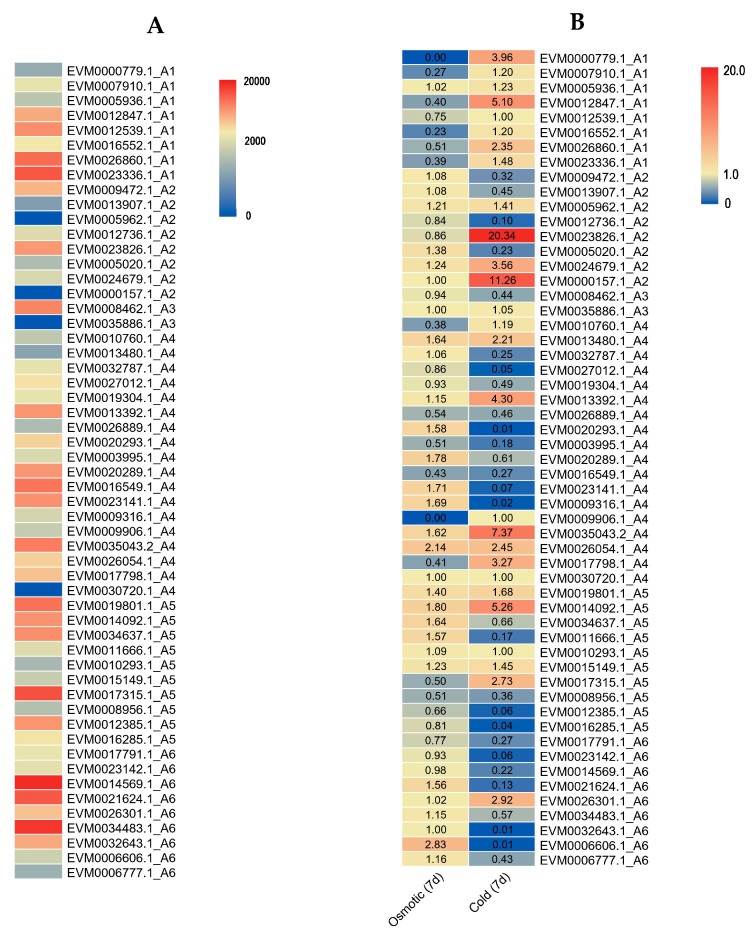
Expression profiles of the DREB subfamily genes calculated using RNA-seq datasets. Transcript per million TPM values of all AnDREB subfamily genes in *A. nanus* leaves under normal conditions (**A**); fold change values of all AnDREB subfamily genes exposed to delayed short-term osmotic stress or cold stress (7d) in *A. nanus* leaves (**B**). The color scale represents low expression with blue and high expression with red.

To evaluate the potential function of DREB subfamily genes, specifically, their roles in environmental stress response, gene expression patterns of DREB subfamily genes in *A. nanus* were investigated using the whole transcriptome sequencing (RNA-seq) datasets and quantitative reverse transcriptase PCR (qRT-PCR) analysis. First, RNA-seq datasets (NCBI SRA accession numbers SRR11089024 to SRR11089029 and SRR11087599 to SRR11087604) were used to quantitate the expression level of DREB subfamily genes in leaves of *A. nanus* seedlings grown under normal conditions or exposed to delayed short-term treatment (7d) of cold or osmotic stress (Figure 10). The expression levels of 55 DREB subfamily members in unstressed leaves were highly variable; several genes exhibited high expression levels, such as *EVM0014569.1*, *EVM0034483.1*, *EVM0017315.1*, and *EVM0026860.1*, whereas some genes displayed very low level or were hard to detect, including *EVM0000157.1*, *EVM0005962.1*, *EVM0013480.1*, *EVM0013907.1*, *EVM0030720.1*, and *EVM0035886.1*.

After delayed short-term osmotic stress or cold stress (7d), the expression of many DREB subfamily members changed significantly. *EVM0026054.1* and *EVM0014092.1* were upregulated, and *EVM0003995.1* and *EVM0016549.1* were downregulated under both stresses, whereas genes such as *EVM0023826.1* and *EVM0012847.1* were induced under cold stress, and were downregulated or did not exhibit significant change under osmotic stress.

**Figure 11:**
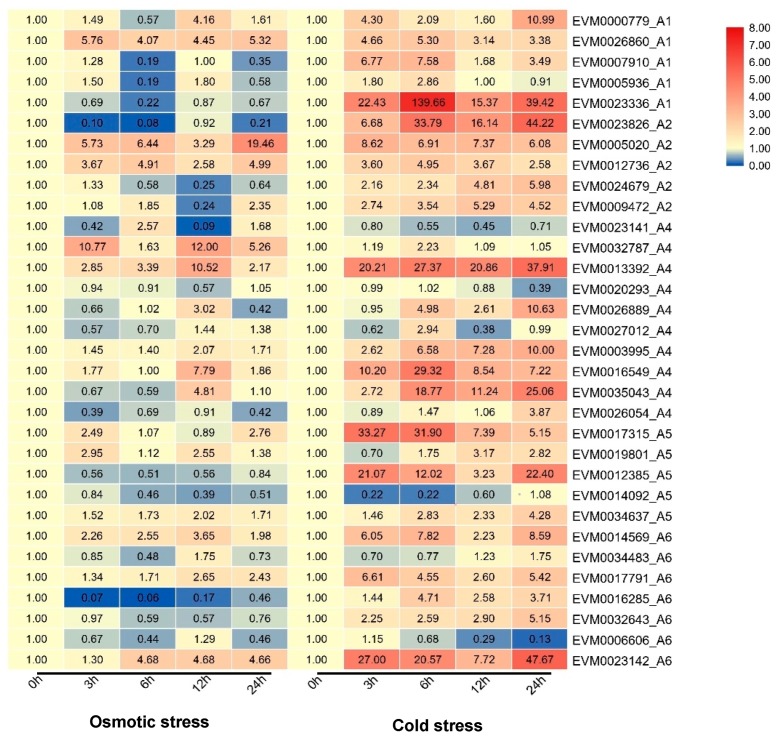
Expression profiles of 32 DREB subfamily genes under 3 h, 6 h, 12 h, and 24 h of osmotic and cold treatment in *A. nanus* leaves. Gene expression levels were quantified through quantitative reverse transcriptase PCR (qRT-PCR) analysis and the experimental values were normalized using eIF1 as the reference gene.

To reveal the dynamic expression patterns of DREB subfamily genes under short-term osmotic and cold stresses, qRT-PCR analyses were conducted using *A. nanus* seedlings exposed to 3 h, 6 h, 12 h, and 24 h of cold or osmotic stress treatment (Figure 11). A total of 32 pairs of primers were used in qRT-PCR analysis, and we were unable to obtain effective primers for some other DREB genes, including the 6 DREB genes with very low abundance in leaves (i.e., *EVM0000157.1*, *EVM0005962.1*, *EVM0013480.1*, *EVM0013907.1*, *EVM0030720.1*, and *EVM0035886.1*).

Approximately half of the DREB subfamily genes (56.25%) were upregulated more than 2-fold under short-term osmotic stress, whereas under short-term cold stress, the percentage rose to 84.38%. This phenomenon is also observed in the RNA-seq analysis results, indicating that, in general, the response of DREB subfamily genes to cold stress is significantly greater than that of osmotic stress. 

Specifically, EVM0026860.1, EVM0005020.1, EVM0012736.1, EVM0032787.1, EVM0013392.1, EVM0014569.1, and EVM0023142.1 were strongly upregulated by osmotic stress treatment, and EVM0023336.1, EVM0023826.1, EVM0013392.1, EVM0016549.1, EVM0035043.1, EVM0012385.1, and EVM0023142.1 were strongly upregulated by cold stress treatment. We also noted that seven genes were upregulated under both osmotic and cold stress treatment, including EVM0026860.1, EVM0005020.1, EVM0012736.1, EVM0013392.1, EVM0016549.1, EVM0014569.1, and EVM0023142.1. These DERB family genes may play essential roles in the response to low-temperature and drought stress in *A. nanus*.

## 3. Discussion

High-quality whole genome sequences of *A. nanus* enabled the genome-wide identification of AP2-ERF family member genes and a comparison with other plant species. Both the total number of AnAP2/ERF families and the quantity of DREB subfamily members in *A. nanus* were similar to those of some leguminous plants such as *P. vulgaris* and *C. cajan*, and a little higher than those of some other leguminous plants including *G. max*, *M. truncatula*, and *C. arietinum* (Figure 1). Of the five subfamilies of the AP2/ERF family in *A. nanus*, the ERF subfamily has the largest number of members, followed by the DREB and AP2 subfamilies. Such a distribution pattern was also observed in other plants such as *P. vulgaris* (Figure 1). It is noteworthy that the number of DREB subfamily members in *A. nanus* is larger than those of the other leguminous plants (Table S6), which may be associated with the high number of group A1, A2, and A6 of the *A. nanus* DREB subfamily. In addition, we observed that the quantity of members in the B1 group is significantly higher than those of the other leguminous plants (Table S6). Whether these observations are associated with the abiotic stress tolerance of *A. nanus* needs further investigation.

Motifs identified in transcription factors can be involved in transcriptional activity, protein–protein interactions, and nuclear localization [7]. In the present study, 20 motifs were identified from the AnAP2/ERF family transcription factors. Motif 1~5 fell in the AP2/ERF domain, motifs 1, 2, and 5 were present in all family members, and motifs 3 and 4 were only found in the AP2 subfamily. The other motifs were located outside the AP2/ERF domain. Of them, the LNFP motif in motifs 1, 6, and 14 was DREB subfamily-specific, which were known to be related to disease resistance [40]. LPR[P/A] motif in motif 6 has been identified as essential signatures for calcineurin B-like (CBL)-interacting serine/threonine-protein kinase 12 [41]. Some motifs were present in a group-specific manner; for example, motif 20 was only detected in the DREB A1 group and motif 14 was only found in the DREB A6 group. 

Gene duplications are suggested to be one of the primary driving forces in the evolution of genomes, and segmental duplication, tandem duplication, and transposition events, including retroposition and replicative transposition, are three principal evolutionary pathways to generate duplicated genes. Of the three pathways, segmental and tandem duplications are wide-spread events in plant genomes and play important roles in the evolution of gene families and adaptation to environmental changes in plants [42]. Of the 174 AnAP2/ERF family genes, 63.22% (110/174) were present in segmental or tandem duplication, suggesting that tandem and segmental duplication represent two principal pathways for gene expansion of the AP2/ERF family in *A. nanus*. Of the 174, 27 (15.52%) AP2/ERF subfamily genes in *A. nanus* were tandemly duplicated genes, whereas up to 47.70% (83/174) were segmentally duplicated genes. This quantity of segmentally duplicated genes was significantly higher than that of tandem-duplicated genes in the AP2/ERF family in *A. nanus*, and similar results have been reported in other plant species such as cucumber [43] and common bean [21]. The observation supported the hypothesis that, compared with tandem duplication, segmental duplication made a major contribution to the expansion of the AP2/ERF gene family in *A. nanus*. However, all tandem-duplicated genes in the AP2/ERF family in *A. nanus* belonged to the DREB and ERF subfamilies, indicating tandem duplication also played significant roles in the expansion of the DREB and ERF subfamilies.

Promoter analysis provided useful information to understand the upstream transcription factors that govern the tightly controlled regulation of DREB genes. In the past two decades, a large number of research studies on the regulation of DREB genes have revealed a complex network of different transcription factors involved in their regulation, and the DREB promoters have been proposed to be the central hubs that integrate multiple environmental and internal developmental signals [44]. Our results showed that promoter regions of AnAP2/ERF genes contained a certain number of MYB, MYC, and WRKY binding elements, suggesting that certain MYB, MYC, and WRKY transcription factors can regulate the expression of the AP2/ERF family genes in *A. nanus*. 

Among the predicted *cis*-acting elements in DREB subfamily genes in *A. nanus*, MYC/ICE1 binding site (CANNTG, the canonical recognition sequence for bHLH family transcription factors) was one of the most frequently occurring ones. Nearly all of the genes carried the MYC/ICE1 binding site except *EVM0011666.1*, and 44 of the DREB genes (80.0%) carried more than six copies of the MYC/ICE1 binding site, indicating that this *cis*-acting element may play an important role in the regulation of DREB genes in *A. nanus*. The expression patterns of DREB genes under cold and osmotic stresses were consistent with the results of promoter analyses, and the large number of MYC/ICE1 binding sites in the promoters of DREB genes may mediate the induction of DREB subfamily genes under low-temperature and drought stress by interacting with ICE1 transcription factors in *A. nanus*. Previous research has shown that the overexpression of ICE1 enhanced the induction of DREB genes and their target genes, consequently increasing the levels of soluble sugars, and late embryogenesis abundant (LEA) proteins, and finally enhancing tolerance to osmotic and cold stresses in tobacco [45]. Although all cold-induced DREB genes contain multiple copies of the MYC/ICE1 binding site, the copy number of MYC/ICE1 binding sites in the promoters of DREB genes was not positively correlated with the fold change of the expression level. 

Another frequently occurring *cis*-acting element in promoters of DREB subfamily genes in *A. nanus* is W-box (TTGAC, the binding site of WRKY family transcription factors), which is found in promoters of 50 (90.91%) DREB genes in *A. nanus* (Figure 6). Considering the versatile biological function of WRKY family transcription factors [46], *A. nanus* DREB subfamily genes may respond to environmental signals via regulation by WRKY transcription factors. 

Some AP2/ERF family genes are arranged in clusters on the chromosome and they may be amplified via tandem duplication. These proteins have similar protein sequences, but we also want to know the evolutionary relationship between them and whether they have similar expression patterns under stress conditions. One of the largest clusters (cluster 10) contained 4 DREB genes, and their order is *EVM0035043.2*, *EVM0026054.1*, *EVM0017798.1*, and *EVM0023336.1*. The first three genes are members of group A4 and the last one is a member of group A1. Phylogenetic analysis showed that DREB A1 and A4 belong to the same large branch, and A1 is a branch of A4 (Figure 3). *EVM0023336.1* is located in the branch of group A1, while *EVM0035043.2*, *EVM0026054.1*, and *EVM0017798.1* form an independent small branch in branch A4. The multiple sequence alignment of AP2/ERF conservative domains of the DREB subfamily is consistent with the above observations (Figure S1). We further speculate that the four DREB genes in cluster 10 may have evolved from two ancestor genes, and that *EVM0035043.2*, *EVM0026054.1*, and *EVM0017798.1* originated from the same ancestor gene. Among the three genes, *EVM0026054.1* and EVM0017798.1 are more similar in amino acid sequence, which may be derived from an ancestor gene that appears later. In addition, these four genes exhibited a considerable amount of expression in the leaves, and were upregulated under low temperature.

Another large tandem duplication gene cluster is cluster 8, which consists of *EVM0020169.1*, *EVM0004125.1*, *EVM0000808.1*, and *EVM0021771.1*, four genes belonging to the ERF subfamily B3 group. Phylogenetic analysis shows that the ERF subfamily B3 group consists of three branches; *EVM0020169.1* is located in a branch, and *EVM0004125.1*, *EVM0000808.1* and *EVM0021771.1* form a small independent clade in another branch. Although *EVM0020169.1*, *EVM0004125.1*, *EVM0000808.1*, and *EVM0021771.1* form a gene cluster on chromosome 4, *EVM0028015.1*, which is most similar to *EVM0020169.1* in sequence and also belongs to ERF subfamily B3 group, is located on chromosome 9. 

Both qRT-PCR analysis and RNA-seq datasets were used to evaluate the gene expression level of DREB genes under cold and osmotic stresses, and a collection of cold stress-responsive or osmotic stress-responsive DREB genes were identified. These highly induced DERB genes under cold and osmotic stresses may contribute greatly to the abiotic stress response of *A. nanus*, and thus deserve to be selected for further functional investigation by ectopic expression in a model plant such as *Arabidopsis*. Although there is no report on the transgenic functional study of DREB genes in *A. nanus*, the functional analyses of two DREB genes in *A. mongolicus*, a relative plant of *A. nanus*, supported our expression analysis results. *AmDREB2C*, the ortholog of *EVM0017315.1*, and *AmDREB3*, the ortholog of *EVM0023826.1*, were cloned, and their expression patterns and biological functions were studied [47,48]. *AmDREB3* was shown to be induced by drought, salt, heat, cold, and abscisic acid treatments [48], and *AmDREB2C* exhibited the highest expression levels in winter in seedlings in the field, and its expression was induced by cold, heat, and drought stresses in laboratory-cultured seedlings [47]. The orthologs in *A. nanus*, *EVM0017315.1* and *EVM0023826.1*, were also highly induced by cold stress (Figure 10 and Figure 11). The transgenic *Arabidopsis* overexpressing the *AmDREB2C* and *AmDREB3* enhances the tolerance to abiotic stress such as freezing, heat, and drought, indicating that the strategy of mining stress-resistant genes from a stress-resistant plant such as *Ammopiptanthus* is effective. In brief, the expression profiling of the AP2/ERF family genes under low-temperature and drought stress will be helpful in understanding the biological functions of the individual AP2/ERF transcription factors. 

## 4. Materials and Methods 

### 4.1. Identification of Putative AP2/ERF Transcription Factors in *A. nanus*

Genome sequence data were obtained from the *A. nanus* genome project [32]. The hidden Markov model (HMM) model of the Apetala 2 (AP2) (PF00847) was downloaded from the Pfam database [49], and the AP2/ERF family TFs in *A. nanus* were identified using HMMER3 (v. 3.0) software [50] with a defined threshold of E < 1e^−5^. The NCBI Conserved Domain Search Service (CD Search) [51] was used to confirm manually the predicted AP2/ERF family TFs.

### 4.2. Multiple Alignment and Phylogenetic Analysis

Multiple sequence alignment of the AP2/ERF family TFs was conducted using ClustalW [52]. The phylogenetic trees were constructed with MEGA-X software [53] using the neighbor-joining (NJ) method. The phylogenetic trees were tested using bootstrapping with 2000 replicates. The AP2/ERF family databases of *Arabidopsis* were downloaded from the plant transcription factor database [54].

### 4.3. Identification of Conserved Domains

Conserved domains were predicted using the tool DIALIGN-PFAM [55]. The deduced amino acid sequences of AP2/ERF genes were analyzed with the MEME (Multiple Em for Motif Elicitation) suite 5.0.1 [36] using the following parameters: optimum width 10~200 amino acids of a motif, and the maximum number of motifs set at 20.

### 4.4. Visualization of AP2/ERF Family Genes on Chromosomes

Diagrams of the chromosome locations of all AP2/ERF genes in *A. nanus* were visualized using MapChart V2.3 [56].

### 4.5. Prediction of *Cis*-acting Elements in Promoter Regions of Genes

The software PLACE [57] was used to locate the putative *cis*-acting elements in the promoter regions of the DREB subfamily genes. Additionally, 2000 bp DNA sequence upstream of the start codon of each DREB gene were used for *cis*-acting element prediction.

### 4.6. Gene Duplication and Divergence Analysis 

Tandem duplication means the generation of tandem gene arrays consisting of identical sequences in close genomic proximity. In the present study, the genes present in tandem duplication were identified using a criterion described previously [58], and tandem-duplicated genes were defined as adjacent homologous genes on a single chromosome, with no more than one intervening gene. The paralogous genes of identified AP2/ERF genes of *A. nanus* were investigated using MCScanX software [59] and visualized using the program Circos [60]. The divergence between homologous genes and the selective pressure on duplicated genes were estimated by calculating synonymous (Ks) and non-synonymous substitutions (Ka) per site between the duplicated gene pairs using KaKsAnalysis in the PlantTribes collection [61].

### 4.7. Plant Materials and Stress Treatments

The seeds of *A. nanus* were collected from the arid areas in Wuqia county, Xinjiang Autonomous Region, China. After surface sterilization with 70% (*v*/*v*) ethanol, the seeds were planted in 30 cm diameter pots containing a 3:1 (*v*/*v*) mixture of perlite and vermiculite. *A. nanus* seedlings were grown in a plant incubator under 120 μmol m^−2^ s^−1^ photosynthetic photon flux density, with a photoperiod of 16 h, at around 25 °C and a relative humidity of 35%. The seedlings were irrigated every four days with a solution of half-strength Hoagland. 

Eight weeks after germination, seedlings with similar height were exposed to cold and osmotic stress treatments. The seedlings were randomly split into nine groups. Four osmotic stress-treated groups were watered with 20% PEG-6000 for 3 h, 6 h, 12 h, or 24 h, four cold stress-treated groups were moved to a growth chamber at 4 °C for 3 h, 6 h, 12 h, or 24 h, and the untreated seedlings were used as the control. Leaf samples were collected from seedlings of the control and stress-treated groups, snap-frozen in nitrogen, and stored at −80 °C until use. 

### 4.8. qRT-PCR Analysis of the DREB Subfamily Genes in *A. nanus*

RNA extraction was conducted using the Trizol reagent according to the user’s guide (Invitrogen, Carlsbad, CA, USA). Quantitative reverse transcriptase PCR (qRT-PCR) analysis was performed following a method described earlier [62]. Three biological replicates were collected for each group and at least three technical repeats for each biological replicate were assayed. The relative gene expression was calculated using the 2^−ΔΔCt^ method [63]. The gene expression levels were normalized against an internal reference gene, eukaryotic translation initiation factor 1 (eIF1). Oligo 7 [64] was used to design the primer pairs for qRT-PCR analysis of the DREB subfamily genes, and all primer pairs used in the current study are listed in Table S7. 

### 4.9. Statistics

Student *t*-tests were used to determine significance, and the probability level of *p* ≤ 0.05 was set for statistical significance. Genes were deemed to be differentially expressed if they demonstrated a fold change of at least 2 and a *p*-value less than 0.05.

## 5. Conclusions

In the current study, 174 AP2/ERF family genes were identified from the genome sequence of *A. nanus*, and these genes were further divided into five subfamilies, including ERF, DREB, AP2, RAV, and Soloist. Gene expansion occurred in the DREB subfamily and ERF subfamily B1 group, and the major gene amplification mode was segmental duplication, followed by tandem duplication. Multiple sequence alignment validated the conservation of the AP2 domains of AP2/ERF family TFs and revealed the characteristics of the AP2 domains of different subfamilies. *Cis*-acting element prediction indicated that MYB, ICE1 and WRKY TFs might regulate the expression of DREB genes in *A. nanus*. Gene profiling identified a collection of cold or osmotic stress-induced genes in the AP2/ERF family. The highly stress-induced DREB genes identified in the present study, including *EVM0023336.1*, *EVM0013392.1*, and *EVM0026860.1*, may contribute to the stress tolerance of *A. nanus*.

## Supplementary Materials

The following are available online at https://www.mdpi.com/2223-7747/9/4/455/s1, Figure S1: Comparison of deduced amino acid sequences of the AP2 domain of the DREB subfamily proteins from *A. thaliana* (At) and *A. nanus* (An), Figure S2: Comparison of deduced amino acid sequences of the AP2 domain of the ERF subfamily proteins from *A. thaliana* (At) and *A. nanus* (An), Figure S3: Comparison of deduced amino acid sequences of the AP2 domain and B3 domain of the RAV subfamily proteins from *A. thaliana* (At) and *A. nanus* (An), Figure S4: Comparison of deduced amino acid sequences of the AP2 domain of the AP2 subfamily proteins from *A. thaliana* (At) and *A. nanus* (An), Figure S5: The 20 conserved motifs identified in the AP2/ERF family members, Figure S6. Motif compositions and gene structures of the AP2/ERF family members in *A. nanus*, Table S1: Physical and chemical characterization of AP2/ERF family from *A. nanus*, Table S2: The AP2/ERF family genes with tandem duplication relationships in *A. nanus*, Table S3: AP2/ERF family genes with segmental duplication relationships in *A. nanus*, Table S4: The Ka/Ks ratios for the paralogous gene pairs in the AP2/ERF gene family in *A. nanus*, Table S5: The Ka/Ks ratios for the orthologous gene pairs in the AP2/ERF family between *A. nanus* and *G. max*. Table S6: Member numbers of each subfamily and group in the AP2/ERF family in *A. nanus* and other legume species, Table S7: The primers used in qRT-PCR analysis.
