# Supplementary material for: Characterization of the AP2/ERF Transcription Factor Family and Expression Profiling of DREB Subfamily under Cold and Osmotic Stresses in Ammopiptanthus nanus
Source: Plants (Basel). 2020 Apr 4;9(4):455. doi: 10.3390/plants9040455 (PMC7238250; doi:10.3390/plants9040455)
Supplement: Supplementary file 1 [file plants-09-00455-s001.zip › plants-735736-proofed-supplementary/supplementary_files_0331.docx]

**Table S1.** Physical and chemical characterization of the AP2/ERF family proteins from *Ammopiptanthus nanus*

| **Number** | **Sequence ID** | **Subfamily** | **Group** | **Genome location** | **CDs length (bp)** | **Amino acid length** | **Number of introns** | **pI** | **Mw (Kda)** |
| --- | --- | --- | --- | --- | --- | --- | --- | --- | --- |
| 1 | EVM0012847.1 | DREB | A1 | Chr 2: 410713--4108766 | 1631 | 244 | 0 | 6.39 | 27824.66 |
| 2 | EVM0012539.1 | DREB | A1 | Chr 2: 4114932--4116193 | 1262 | 230 | 0 | 7.26 | 26251.98 |
| 3 | EVM0016552.1 | DREB | A1 | Chr 2: 90849097--90849861 | 765 | 254 | 0 | 5.33 | 28330.01 |
| 4 | EVM0026860.1 | DREB | A1 | Chr 3: 60762847--60763448 | 602 | 182 | 1 | 6.01 | 20949.37 |
| 5 | EVM0007910.1 | DREB | A1 | Chr 3: 777994040--77800261 | 858 | 285 | 0 | 6.36 | 31259.04 |
| 6 | EVM0005936.1 | DREB | A1 | Chr 4: 6194139--61941961 | 567 | 188 | 0 | 8.58 | 21205.62 |
| 7 | EVM0023336.1 | DREB | A1 | Chr 4: 81725315--81727040 | 1726 | 216 | 0 | 5.74 | 23953.67 |
| 8 | EVM0000779.1 | DREB | A1 | Chr 7: 8765610--8766380 | 771 | 256 | 0 | 6.19 | 28269.85 |
| 9 | EVM0000157.1 | DREB | A2 | Chr 3: 15355336--15357775 | 2440 | 325 | 6 | 7.15 | 36381.54 |
| 10 | EVM0024679.1 | DREB | A2 | Chr 3: 2109735--2112169 | 2435 | 543 | 1 | 5.99 | 60195.84 |
| 11 | EVM0009472.1 | DREB | A2 | Chr 4: 70623729--70624660 | 932 | 245 | 1 | 8.67 | 27195.98 |
| 12 | EVM0023826.1 | DREB | A2 | Chr 5: 7155336--7157742 | 2407 | 396 | 0 | 4.67 | 43771.37 |
| 13 | EVM0013907.1 | DREB | A2 | Chr 6: 3549257--3550048 | 792 | 263 | 0 | 5.00 | 29171.33 |
| 14 | EVM0005962.1 | DREB | A2 | Chr 7: 74773744--74774606 | 863 | 257 | 0 | 5.26 | 27978.46 |
| 15 | EVM0005020.1 | DREB | A2 | Chr 8: 46544675--46554932 | 10258 | 516 | 2 | 5.82 | 58105.08 |
| 16 | EVM0012736.1 | DREB | A2 | Chr 9: 63953510--63954358 | 849 | 282 | 0 | 5.62 | 31662.58 |
| 17 | EVM0035886.1 | DREB | A3 | Chr 5: 7326568--7327744 | 1177 | 336 | 1 | 7.25 | 36857.60 |
| 18 | EVM0008462.1 | DREB | A3 | Chr 6: 85555470--85558101 | 2632 | 347 | 1 | 6.11 | 38435.20 |
| 19 | EVM0009316.1 | DREB | A4 | Chr 1: 1730341--1730931 | 591 | 196 | 0 | 5.62 | 21403.33 |
| 20 | EVM0010760.1 | DREB | A4 | Chr 2: 2385270--2386073 | 804 | 191 | 0 | 4.97 | 21575.59 |
| 21 | EVM0013480.1 | DREB | A4 | Chr 2: 2431657--2432235 | 579 | 192 | 0 | 5.73 | 21289.41 |
| 22 | EVM0020289.1 | DREB | A4 | Chr 2: 5316685--5318164 | 1480 | 215 | 0 | 5.27 | 23730.63 |
| 23 | EVM0020293.1 | DREB | A4 | Chr 2: 89233643--89235835 | 2193 | 222 | 1 | 5.50 | 24861.16 |
| 24 | EVM0009906.1 | DREB | A4 | Chr 2: 90869192--90869746 | 555 | 184 | 0 | 5.19 | 20014.58 |
| 25 | EVM0026889.1 | DREB | A4 | Chr 2: 94448510--94449439 | 930 | 233 | 0 | 5.75 | 25576.41 |
| 26 | EVM0003995.1 | DREB | A4 | Chr 3: 12270369--12271487 | 1119 | 275 | 0 | 5.99 | 29952.38 |
| 27 | EVM0016549.1 | DREB | A4 | Chr 3: 89051891--89052919 | 1029 | 187 | 0 | 6.30 | 20770.24 |
| 28 | EVM0035043.2 | DREB | A4 | Chr 4: 81672628--81674306 | 1679 | 213 | 1 | 6.70 | 23233.37 |
| 29 | EVM0026054.1 | DREB | A4 | Chr 4: 81686994--81687647 | 654 | 217 | 0 | 4.70 | 23007.02 |
| 30 | EVM0017798.1 | DREB | A4 | Chr 4: 81709436--81710143 | 708 | 235 | 0 | 4.93 | 25036.15 |
| 31 | EVM0023141.1 | DREB | A4 | Chr 5: 18404558--18405245 | 688 | 174 | 0 | 6.90 | 18915.27 |
| 32 | EVM0019304.1 | DREB | A4 | Chr 6: 10460253--10461307 | 1055 | 226 | 0 | 5.15 | 25200.32 |
| 33 | EVM0032787.1 | DREB | A4 | Chr 7: 2819916--2820891 | 976 | 212 | 0 | 6.92 | 23650.83 |
| 34 | EVM0013392.1 | DREB | A4 | Chr 7: 80717998--80719497 | 1500 | 192 | 1 | 5.87 | 21491.54 |
| 35 | EVM0027012.1 | DREB | A4 | Chr 9: 5529913--5531626 | 1714 | 213 | 1 | 5.44 | 24054.82 |
| 36 | EVM0030720.1 | DREB | A4 | Chr 9:66339248--66339823 | 576 | 191 | 0 | 4.55 | 20015.76 |
| 37 | EVM0016285.1 | DREB | A5 | Chr 2: 2519018--2519908 | 891 | 174 | 0 | 6.29 | 19661.54 |
| 38 | EVM0034637.1 | DREB | A5 | Chr 2: 8212596--8213579 | 984 | 161 | 0 | 8.97 | 17841.91 |
| 39 | EVM0011666.1 | DREB | A5 | Chr 2: 84823859--84824713 | 855 | 157 | 1 | 10.12 | 17373.09 |
| 40 | EVM0010293.1 | DREB | A5 | Chr 5: 15154697--15155377 | 681 | 226 | 0 | 4.72 | 24647.70 |
| 41 | EVM0015149.1 | DREB | A5 | Chr 6: 14858799--14859714 | 916 | 231 | 0 | 5.19 | 25240.93 |
| 42 | EVM0019801.1 | DREB | A5 | Chr 6: 5102989--5104203 | 1215 | 176 | 0 | 5.96 | 20082.81 |
| 43 | EVM0008956.1 | DREB | A5 | Chr 6: 7172719--7173713 | 995 | 232 | 0 | 4.60 | 25820.24 |
| 44 | EVM0012385.1 | DREB | A5 | Chr 7: 67821153--67822336 | 1184 | 236 | 0 | 4.40 | 25926.02 |
| 45 | EVM0014092.1 | DREB | A5 | Chr 7: 72331031--72333106 | 2076 | 158 | 0 | 9.76 | 17916.98 |
| 46 | EVM0017315.1 | DREB | A5 | Chr 8: 71559856--71560924 | 1069 | 212 | 0 | 5.18 | 24076.58 |
| 47 | EVM0032643.1 | DREB | A6 | Chr 2: 2918590--2920292 | 1703 | 326 | 0 | 8.68 | 35720.65 |
| 48 | EVM0006606.1 | DREB | A6 | Chr 2: 92769158--92770769 | 1612 | 300 | 0 | 8.49 | 33399.40 |
| 49 | EVM0023142.1 | DREB | A6 | Chr 3: 74398509--74401302 | 2794 | 425 | 0 | 5.62 | 48596.10 |
| 50 | EVM0006777.1 | DREB | A6 | Chr 4: 66395928--66397373 | 1446 | 402 | 0 | 8.68 | 43892.09 |
| 51 | EVM0014569.1 | DREB | A6 | Chr 5: 14325067--14326862 | 1796 | 353 | 0 | 6.19 | 39174.12 |
| 52 | EVM0021624.1 | DREB | A6 | Chr 6: 13868721--13870871 | 2151 | 360 | 0 | 6.56 | 39608.41 |
| 53 | EVM0034483.1 | DREB | A6 | Chr 6: 86624548--86626344 | 1797 | 370 | 1 | 5.48 | 41939.23 |
| 54 | EVM0017791.1 | DREB | A6 | Chr 7: 11801724--11804356 | 2633 | 421 | 1 | 5.49 | 47358.70 |
| 55 | EVM0026301.1 | DREB | A6 | Chr 8: 58763756--58765475 | 1720 | 308 | 0 | 6.10 | 34491.03 |
| 56 | EVM0019855.1 | ERF | B1 | Chr 1: 70578967--70581212 | 2246 | 238 | 1 | 9.00 | 25758.82 |
| 57 | EVM0007953.1 | ERF | B1 | Chr 1: 96646860--96647840 | 981 | 293 | 1 | 5.36 | 32723.28 |
| 58 | EVM0016025.1 | ERF | B1 | Chr 1: 98221726--98223374 | 1649 | 335 | 0 | 5.64 | 35962.11 |
| 59 | EVM0019650.1 | ERF | B1 | Chr 1: 99562643--99563859 | 1217 | 196 | 0 | 6.31 | 21267.71 |
| 60 | EVM0032083.1 | ERF | B1 | Chr 1: 99665940--99666819 | 880 | 168 | 0 | 9.66 | 18095.40 |
| 61 | EVM0008621.1 | ERF | B1 | Chr 2: 17423862--17425040 | 1179 | 392 | 0 | 6.25 | 43610.64 |
| 62 | EVM0029775.1 | ERF | B1 | Chr 2: 21637980--21639209 | 1230 | 409 | 0 | 6.28 | 45960.24 |
| 63 | EVM0000501.1 | ERF | B1 | Chr 2: 25379979--25380949 | 971 | 311 | 1 | 4.96 | 34980.86 |
| 64 | EVM0002488.1 | ERF | B1 | Chr 2: 31579068--31580107 | 1040 | 162 | 0 | 9.37 | 17851.87 |
| 65 | EVM0012229.1 | ERF | B1 | Chr 3: 55123872--55124300 | 429 | 142 | 0 | 5.12 | 15789.96 |
| 66 | EVM0024664.1 | ERF | B1 | Chr 3: 70313985--70315450 | 1466 | 139 | 2 | 9.80 | 15428.82 |
| 67 | EVM0004053.1 | ERF | B1 | Chr 3: 73798039--73799003 | 965 | 235 | 0 | 9.33 | 25771.74 |
| 68 | EVM0008586.1 | ERF | B1 | Chr 4: 17995510--17996511 | 1002 | 268 | 0 | 6.45 | 30204.80 |
| 69 | EVM0028570.1 | ERF | B1 | Chr 4: 5660767--5662078 | 1312 | 409 | 0 | 4.53 | 45867.75 |
| 70 | EVM0025961.1 | ERF | B1 | Chr 4: 65580510--65582620 | 2111 | 368 | 4 | 4.39 | 41415.30 |
| 71 | EVM0026653.1 | ERF | B1 | Chr 4: 79269681--79270112 | 432 | 143 | 0 | 6.23 | 16524.52 |
| 72 | EVM0007852.1 | ERF | B1 | Chr 5: 21529848--21531114 | 1267 | 308 | 0 | 8.25 | 33283.21 |
| 73 | EVM0019498.1 | ERF | B1 | Chr 5: 2676698--2679323 | 2626 | 231 | 0 | 7.09 | 24972.29 |
| 74 | EVM0035397.1 | ERF | B1 | Chr 5: 6679229--6680256 | 1028 | 209 | 0 | 6.52 | 23187.34 |
| 75 | EVM0031383.1 | ERF | B1 | Chr 5: 74870938--74872128 | 1191 | 358 | 2 | 4.80 | 40649.51 |
| 76 | EVM0003974.1 | ERF | B1 | Chr 7: 1100435--1101001 | 567 | 188 | 0 | 10.13 | 20169.32 |
| 77 | EVM0018613.1 | ERF | B1 | Chr 7: 12342458--12343513 | 1056 | 235 | 0 | 8.52 | 26133.16 |
| 78 | EVM0003729.1 | ERF | B1 | Chr 7: 19440883--19442060 | 1178 | 272 | 0 | 6.01 | 30438.03 |
| 79 | EVM0017089.1 | ERF | B1 | Chr 7: 21526627--21528806 | 2180 | 278 | 1 | 5.89 | 30979.81 |
| 80 | EVM0026051.1 | ERF | B1 | Chr 8: 56024248--56025009 | 762 | 253 | 0 | 7.59 | 28855.95 |
| 81 | EVM0016298.1 | ERF | B1 | Chr 8: 65520879--65522028 | 1150 | 206 | 0 | 8.88 | 23172.47 |
| 82 | EVM0013260.1 | ERF | B1 | Chr 8: 74065934--74067016 | 1083 | 360 | 0 | 4.95 | 39656.90 |
| 83 | EVM0026627.1 | ERF | B1 | Chr 9: 60028929--60030223 | 1295 | 210 | 0 | 9.68 | 22733.52 |
| 84 | EVM0026047.2 | ERF | B2 | Chr 1: 487496--490792 | 3297 | 376 | 1 | 5.03 | 41622.20 |
| 85 | EVM0036970.1 | ERF | B2 | Chr 1: 89087844--89089888 | 2045 | 245 | 1 | 6.31 | 27277.74 |
| 86 | EVM0026727.1 | ERF | B2 | Chr 2: 25707531--25708353 | 823 | 135 | 1 | 9.47 | 15179.78 |
| 87 | EVM0024520.1 | ERF | B2 | Chr 4: 75118983--75120668 | 1686 | 315 | 1 | 8.66 | 35474.71 |
| 88 | EVM0014312.1 | ERF | B2 | Chr 5: 16611321--16614715 | 3395 | 398 | 1 | 5.02 | 43881.55 |
| 89 | EVM0017087.1 | ERF | B2 | Chr 8: 73499574--73501272 | 1699 | 243 | 1 | 8.49 | 27080.78 |
| 90 | EVM0029153.1 | ERF | B2 |  | 769 | 146 | 0 | 5.40 | 16472.11 |
| 91 | EVM0026004.1 | ERF | B3 | Chr 1: 3237521--3238235 | 715 | 213 | 0 | 5.36 | 24154.68 |
| 92 | EVM0019313.1 | ERF | B3 | Chr 1: 99122877--99123941 | 1065 | 286 | 0 | 5.15 | 31930.48 |
| 93 | EVM0031382.1 | ERF | B3 | Chr 2: 29346051--29346740 | 690 | 199 | 1 | 5.11 | 21752.43 |
| 94 | EVM0025097.1 | ERF | B3 | Chr 2: 31575409--31577524 | 2116 | 222 | 0 | 8.28 | 24545.38 |
| 95 | EVM0018957.1 | ERF | B3 | Chr 2: 33605161--33605634 | 474 | 157 | 0 | 5.93 | 17396.70 |
| 96 | EVM0001259.1 | ERF | B3 | Chr 2: 5476350--5477811 | 1462 | 295 | 0 | 8.85 | 33468.20 |
| 97 | EVM0029432.1 | ERF | B3 | Chr 2: 5516388--5518793 | 2406 | 267 | 0 | 8.12 | 29852.97 |
| 98 | EVM0027510.1 | ERF | B3 | Chr 2: 88974996--88976524 | 1529 | 264 | 0 | 5.69 | 28537.18 |
| 99 | EVM0021979.1 | ERF | B3 | Chr 2: 89001375--89002505 | 1131 | 314 | 0 | 5.30 | 35567.17 |
| 100 | EVM0037078.1 | ERF | B3 | Chr 3: 11491977--11493118 | 1142 | 222 | 0 | 9.42 | 25075.56 |
| 101 | EVM0003547.1 | ERF | B3 | Chr 3: 38860086--38862034 | 1949 | 222 | 1 | 8.52 | 25059.24 |
| 102 | EVM0013782.1 | ERF | B3 | Chr 3: 47411995--47413872 | 1878 | 207 | 0 | 6.09 | 23378.65 |
| 103 | EVM0011477.1 | ERF | B3 | Chr 3: 47477835--47479382 | 1548 | 285 | 0 | 8.30 | 31719.69 |
| 104 | EVM0033123.1 | ERF | B3 | Chr 4: 5126747--5128432 | 1686 | 135 | 1 | 9.43 | 15295.85 |
| 105 | EVM0020169.1 | ERF | B3 | Chr 4: 54090084--54090536 | 453 | 150 | 0 | 5.80 | 16892.42 |
| 106 | EVM0004125.1 | ERF | B3 | Chr 4: 54141578--54142252 | 675 | 224 | 0 | 5.21 | 25030.39 |
| 107 | EVM0000808.1 | ERF | B3 | Chr 4: 54339121--54340039 | 919 | 219 | 0 | 6.00 | 24408.17 |
| 108 | EVM0021771.1 | ERF | B3 | Chr 4: 54402724--54403480 | 757 | 219 | 0 | 6.00 | 24438.18 |
| 109 | EVM0025785.1 | ERF | B3 | Chr 4: 66321477--66322052 | 576 | 181 | 1 | 5.26 | 20703.12 |
| 110 | EVM0017483.1 | ERF | B3 | Chr 4: 67445339--67445806 | 468 | 155 | 0 | 7.87 | 16740.05 |
| 111 | EVM0036392.1 | ERF | B3 | Chr 4: 67456404--67456823 | 420 | 139 | 0 | 9.30 | 15855.96 |
| 112 | EVM0011840.1 | ERF | B3 | Chr 4: 67511202--67513299 | 2098 | 288 | 2 | 6.47 | 32737.08 |
| 113 | EVM0029797.1 | ERF | B3 | Chr 8: 4493966--4494583 | 618 | 186 | 1 | 9.28 | 20926.42 |
| 114 | EVM0015734.1 | ERF | B3 | Chr 8: 4509963--4510897 | 935 | 228 | 0 | 5.98 | 25667.54 |
| 115 | EVM0010624.1 | ERF | B3 | Chr 8: 4658105--4658925 | 821 | 235 | 0 | 7.94 | 26292.23 |
| 116 | EVM0032149.1 | ERF | B3 | Chr 9: 69930216--69931341 | 1126 | 266 | 0 | 5.00 | 29782.53 |
| 117 | EVM0028015.1 | ERF | B3 | Chr 9: 69964229--69965192 | 964 | 138 | 0 | 5.96 | 15737.72 |
| 118 | EVM0035142.1 | ERF | B3 | Chr 9: 70172889--70173374 | 486 | 161 | 0 | 9.61 | 17928.29 |
| 119 | EVM0032511.1 | ERF | B3 |  | 2567 | 243 | 1 | 5.64 | 27493.32 |
| 120 | EVM0026285.1 | ERF | B4 | Chr 3: 56174241--56176605 | 2365 | 452 | 1 | 6.32 | 48626.44 |
| 121 | EVM0035087.1 | ERF | B4 | Chr 4: 34120770--34122532 | 1763 | 227 | 1 | 5.89 | 24998.96 |
| 122 | EVM0004579.1 | ERF | B4 | Chr 4: 79775672--79778281 | 2610 | 406 | 1 | 5.68 | 43625.96 |
| 123 | EVM0006216.1 | ERF | B4 | Chr 6: 21521067--21523038 | 1972 | 353 | 1 | 6.46 | 39190.24 |
| 124 | EVM0031497.1 | ERF | B4 | Chr 6: 26485611--26487876 | 2266 | 233 | 1 | 9.39 | 25846.56 |
| 125 | EVM0029311.1 | ERF | B4 | Chr 7: 22218734--22221172 | 2439 | 213 | 1 | 6.62 | 23289.26 |
| 126 | EVM0018011.1 | ERF | B4 | Chr 8: 59552433--59554086 | 1654 | 346 | 1 | 9.59 | 37578.33 |
| 127 | EVM0025743.1 | ERF | B4 | Chr 8: 61059161--61062163 | 3003 | 275 | 1 | 5.68 | 30205.64 |
| 128 | EVM0027069.1 | ERF | B5 | Chr 1: 2398893--2399834 | 942 | 313 | 0 | 6.01 | 36780.92 |
| 129 | EVM0028620.1 | ERF | B5 | Chr 1: 81410826--81412775 | 1950 | 263 | 0 | 9.09 | 29348.74 |
| 130 | EVM0010310.1 | ERF | B5 | Chr 2: 3076356--3077613 | 1258 | 321 | 0 | 5.20 | 36026.46 |
| 131 | EVM0024005.1 | ERF | B5 | Chr 2: 92596550--92597741 | 1192 | 332 | 0 | 5.11 | 37682.17 |
| 132 | EVM0012948.1 | ERF | B5 | Chr 5: 10418656--10420625 | 1970 | 286 | 0 | 8.33 | 31903.78 |
| 133 | EVM0022119.1 | ERF | B5 | Chr 6: 17082261--17083922 | 1662 | 348 | 0 | 4.59 | 38951.99 |
| 134 | EVM0011490.1 | ERF | B5 | Chr 8: 12151557--12152462 | 906 | 301 | 0 | 6.09 | 34948.11 |
| 135 | EVM0026028.1 | ERF | B5 | Chr 9: 16423964--16424959 | 996 | 331 | 0 | 5.80 | 37234.68 |
| 136 | EVM0011527.1 | ERF | B6 | Chr 2: 84063753--84064490 | 738 | 245 | 0 | 7.25 | 27530.29 |
| 137 | EVM0034189.1 | ERF | B6 | Chr 2: 95385299--95386257 | 959 | 268 | 1 | 4.66 | 29707.21 |
| 138 | EVM0010333.1 | ERF | B6 | Chr 4: 86538724--86539818 | 1095 | 364 | 0 | 4.84 | 39939.37 |
| 139 | EVM0008941.1 | ERF | B6 | Chr 5: 29917740--29918849 | 1110 | 369 | 0 | 4.68 | 40516.65 |
| 140 | EVM0019590.1 | ERF | B6 | Chr 6: 7862864--7865823 | 2960 | 269 | 3 | 9.28 | 30927.86 |
| 141 | EVM0019301.1 | ERF | B6 | Chr 6: 8461553--8463197 | 1645 | 208 | 1 | 6.33 | 23056.26 |
| 142 | EVM0034272.1 | ERF | B6 | Chr 7: 1037518--1038400 | 883 | 184 | 1 | 7.09 | 21143.49 |
| 143 | EVM0015677.1 | ERF | B6 | Chr 7: 1617733--1618520 | 788 | 184 | 1 | 9.05 | 21074.46 |
| 144 | EVM0004251.1 | ERF | B6 | Chr 7: 79289140--79290063 | 924 | 307 | 0 | 4.81 | 34290.85 |
| 145 | EVM0020800.1 | ERF | B6 | Chr 8: 66585308--66586569 | 1262 | 228 | 1 | 6.00 | 25341.31 |
| 146 | EVM0015416.1 | ERF | B6 | Chr 9: 2415264--2416550 | 1287 | 192 | 1 | 7.79 | 21710.70 |
| 147 | EVM0031318.1 | ERF | B6 | Chr 9: 3384535--3385833 | 1299 | 194 | 1 | 8.48 | 21821.02 |
| 148 | EVM0002664.1 | RAV |  | Chr 4: 48133011--48134637 | 1627 | 364 | 0 | 9.00 | 39906.21 |
| 149 | EVM0036388.1 | RAV |  | Chr 5: 29075612--29077466 | 1855 | 371 | 0 | 9.23 | 40580.38 |
| 150 | EVM0033695.1 | AP2 |  | Chr 2: 16451989--16457190 | 5202 | 518 | 8 | 6.19 | 56600.16 |
| 151 | EVM0027377.1 | AP2 |  | Chr 2: 21389102--21409622 | 20521 | 425 | 9 | 9.32 | 47633.62 |
| 152 | EVM0009457.1 | AP2 |  | Chr 2: 24103935--24106155 | 2221 | 379 | 8 | 9.51 | 42715.16 |
| 153 | EVM0006226.1 | AP2 |  | Chr 2: 7120725--7124770 | 4046 | 549 | 8 | 8.71 | 61907.37 |
| 154 | EVM0019849.1 | AP2 |  | Chr 2: 8244512--8248042 | 3531 | 515 | 9 | 6.14 | 56812.27 |
| 155 | EVM0035983.1 | AP2 |  | Chr 2: 84927455--84931608 | 4154 | 524 | 9 | 6.63 | 57755.78 |
| 156 | EVM0021580.2 | AP2 |  | Chr 2: 86864709--86868886 | 4178 | 662 | 8 | 6.46 | 74341.80 |
| 157 | EVM0021893.1 | AP2 |  | Chr 3: 18767225--18772037 | 4813 | 691 | 8 | 6.17 | 76010.59 |
| 158 | EVM0005801.1 | AP2 |  | Chr 3: 48622614--48625935 | 3322 | 421 | 7 | 8.98 | 46653.13 |
| 159 | EVM0013242.1 | AP2 |  | Chr 3: 66230020--66234227 | 4208 | 399 | 7 | 5.49 | 44997.68 |
| 160 | EVM0010770.1 | AP2 |  | Chr 3: 80249798--80253150 | 3353 | 478 | 9 | 7.12 | 52388.58 |
| 161 | EVM0027608.1 | AP2 |  | Chr 3: 81993912--81996978 | 3067 | 576 | 8 | 6.29 | 63598.12 |
| 162 | EVM0002293.1 | AP2 |  | Chr 4: 4243081--4245932 | 2852 | 374 | 7 | 7.65 | 42425.15 |
| 163 | EVM0033247.1 | AP2 |  | Chr 4: 70197068--70201835 | 4768 | 658 | 8 | 6.23 | 74619.07 |
| 164 | EVM0037148.1 | AP2 |  | Chr 6: 6006542--6011015 | 4474 | 675 | 8 | 6.47 | 74500.50 |
| 165 | EVM0013295.1 | AP2 |  | Chr 6: 78414688--78418929 | 4242 | 364 | 7 | 6.72 | 41427.03 |
| 166 | EVM0019334.1 | AP2 |  | Chr 7: 4799732--4802932 | 3201 | 573 | 8 | 6.03 | 63640.05 |
| 167 | EVM0001347.1 | AP2 |  | Chr 7: 660100--665697 | 5598 | 377 | 7 | 6.51 | 42638.87 |
| 168 | EVM0007559.1 | AP2 |  | Chr 7: 6690550--6695634 | 5085 | 474 | 8 | 8.74 | 52205.37 |
| 169 | EVM0034544.1 | AP2 |  | Chr 7: 70559656--70563370 | 3715 | 705 | 8 | 6.64 | 77735.20 |
| 170 | EVM0021339.1 | AP2 |  | Chr 8: 69755376--69759615 | 4240 | 524 | 8 | 8.21 | 57286.49 |
| 171 | EVM0014070.1 | AP2 |  | Chr 9: 11620881--11624435 | 3555 | 569 | 8 | 5.96 | 63117.22 |
| 172 | EVM0018988.1 | AP2 |  | Chr 9: 63471444--63475930 | 4487 | 428 | 8 | 9.03 | 47299.53 |
| 173 | EVM0032499.1 | AP2 |  | Chr 9: 68289894--68292868 | 2975 | 466 | 9 | 6.17 | 51863.13 |
| 174 | EVM0028339.1 | Soloist |  | Chr 3: 8374731--8381219 | 6489 | 236 | 5 | 9.40 | 26706.63 |

pI: isoelectric point; Mw: molecular weight.

**Table S4.** The Ka/Ks ratios for the paralogous gene pairs in the AP2/ERF gene family in *A. nanus*

| Gene 1 | Gene 2 | Ka | Ks | Ka\Ks |
| --- | --- | --- | --- | --- |
| *EVM0007953.1* | *EVM0013260.1* | 0.1390 | 0.3293 | 0.4220 |
| *EVM0029797.1* | *EVM0015734.1* | 0.0859 | 0.2166 | 0.3968 |
| *EVM0034483.1* | *EVM0026301.1* | 0.2015 | 0.8682 | 0.2321 |
| *EVM0035397.1* | *EVM0019498.1* | 0.3122 | 2.0351 | 0.1534 |
| *EVM0004579.1* | *EVM0026285.1* | 0.2347 | 0.7489 | 0.3134 |
| *EVM0021624.1* | *EVM0014569.1* | 0.1776 | 0.5752 | 0.3087 |
| *EVM0007559.1* | *EVM0010770.1* | 0.1037 | 0.4126 | 0.2514 |
| *EVM0015677.1* | *EVM0031318.1* | 0.1040 | 0.3866 | 0.2691 |
| *EVM0013392.1* | *EVM0026889.1* | 0.1602 | 0.7733 | 0.2072 |
| *EVM0032149.1* | *EVM0032511.1* | 0.1821 | 0.6899 | 0.2639 |
| *EVM0015149.1* | *EVM0010293.1* | 0.0963 | 0.356 | 0.2705 |
| *EVM0001259.1* | *EVM0021979.1* | 0.1775 | 0.8741 | 0.2031 |
| *EVM0035983.1* | *EVM0019849.1* | 0.0761 | 0.5156 | 0.1475 |
| *EVM0027608.1* | *EVM0019334.1* | 0.1126 | 0.5821 | 0.1935 |
| *EVM0036388.1* | *EVM0002664.1* | 0.1802 | 1.1238 | 0.1603 |
| *EVM0000157.1* | *EVM0024679.1* | 0.1107 | 0.2127 | 0.5204 |
| *EVM0026054.1* | *EVM0017798.1* | 0.0706 | 0.1447 | 0.4877 |
| *EVM0012539.1* | *EVM0012847.1* | 0.0076 | 0.0317 | 0.2407 |
| *EVM0017791.1* | *EVM0023142.1* | 0.2454 | 0.9508 | 0.2581 |
| *EVM0034272.1* | *EVM0015416.1* | 0.0820 | 0.3310 | 0.2479 |
| *EVM0000808.1* | *EVM0021771.1* | 0.0021 | 0.0171 | 0.1242 |
| *EVM0027012.1* | *EVM0032787.1* | 0.1967 | 0.9230 | 0.2131 |
| *EVM0026047.2* | *EVM0014312.1* | 0.1570 | 0.5084 | 0.3089 |
| *EVM0021893.1* | *EVM0033247.1* | 0.1715 | 0.6212 | 0.2761 |
| *EVM0020169.1* | *EVM0028015.1* | 0.1600 | 0.5436 | 0.2942 |
| *EVM0025097.1* | *EVM0019313.1* | 0.1457 | 0.4355 | 0.3346 |
| *EVM0021580.2* | *EVM0006226.1* | 0.1654 | 0.4832 | 0.3424 |
| *EVM0027377.1* | *EVM0033695.1* | 0.1591 | 0.6243 | 0.2549 |
| *EVM0008586.1* | *EVM0003729.1* | 0.1093 | 0.3749 | 0.2916 |
| *EVM0017087.1* | *EVM0026727.1* | 0.4716 | 1.3696 | 0.3443 |
| *EVM0037148.1* | *EVM0034544.1* | 0.1119 | 0.5306 | 0.2109 |

**Table S5.** The Ka/Ks ratios for the orthologous gene pairs in the AP2/ERF family between *A. nanus* and *G. max*

| AP2/ERF gene in *A. nanus* | Orthologous gene in *G. max* | Ka | Ks | Ka\Ks |
| --- | --- | --- | --- | --- |
| *EVM0026301.1* | *Glyma.11G239200.1* | 0.2213 | 0.9131 | 0.2424 |
| *EVM0020289.1* | *Glyma.11G035100.1* | 0.3165 | 1.3148 | 0.2407 |
| *EVM0027608.1* | *Glyma.12G056300.1* | 0.0820 | 0.6325 | 0.1297 |
| *EVM0013782.1* | *Glyma.10G119100.1* | 0.2192 | 0.7078 | 0.3097 |
| *EVM0020800.1* | *Glyma.04G147500.1* | 0.1393 | 1.2785 | 0.1090 |
| *EVM0001259.1* | *Glyma.01G206700.1* | 0.1515 | 0.7175 | 0.2112 |
| *EVM0010333.1* | *Glyma.10G274600.1* | 0.1893 | 0.8036 | 0.2355 |
| *EVM0019313.1* | *Glyma.13G233800.1* | 0.1417 | 0.4211 | 0.3366 |
| *EVM0003547.1* | *Glyma.20G203700.1* | 0.2677 | 1.4060 | 0.1904 |
| *EVM0021624.1* | *Glyma.06G105000.1* | 0.1118 | 1.0309 | 0.1085 |
| *EVM0004053.1* | *Glyma.13G236500.1* | 0.3386 | 8.4115 | 0.0403 |
| *EVM0028620.1* | *Glyma.11G199300.1* | 0.2155 | 0.9472 | 0.2276 |
| *EVM0026051.1* | *Glyma.05G157400.1* | 0.1506 | 0.3024 | 0.4981 |
| *EVM0026653.1* | *Glyma.10G219000.1* | 0.3409 | 1.1870 | 0.2872 |
| *EVM0007910.1* | *Glyma.11G188200.1* | 0.2317 | 0.7031 | 0.3295 |
| *EVM0001347.1* | *Glyma.07G021000.1* | 0.1255 | 0.5500 | 0.2282 |
| *EVM0030720.1* | *Glyma.10G066900.1* | 0.2232 | 3.3523 | 0.0666 |
| *EVM0005962.1* | *Glyma.06G028300.1* | 0.1931 | 0.9539 | 0.2024 |
| *EVM0018011.1* | *Glyma.05G186700.1* | 0.2718 | 0.8928 | 0.3044 |
| *EVM0028570.1* | *Glyma.13G060600.1* | 0.0768 | 0.5443 | 0.1410 |
| *EVM0011477.1* | *Glyma.20G070000.1* | 0.2145 | 0.8451 | 0.2538 |
| *EVM0008941.1* | *Glyma.08G261500.1* | 0.2349 | 0.7959 | 0.2951 |
| *EVM0015416.1* | *Glyma.08G216600.1* | 0.0788 | 0.2648 | 0.2975 |
| *EVM0036970.1* | *Glyma.19G213100.1* | 0.5165 | 2.7956 | 0.1848 |
| *EVM0012736.1* | *Glyma.19G142000.1* | 0.0950 | 0.3271 | 0.2905 |
| *EVM0028015.1* | *Glyma.03G162400.1* | 0.2050 | 0.7899 | 0.2595 |
| *EVM0007852.1* | *Glyma.07G078600.1* | 0.1529 | 0.5738 | 0.2665 |
| *EVM0019498.1* | *Glyma.02G294100.1* | 0.1561 | 1.5649 | 0.0998 |
| *EVM0002664.1* | *Glyma.10G204400.1* | 0.1412 | 0.6914 | 0.2043 |
| *EVM0010770.1* | *Glyma.12G073300.1* | 0.1037 | 0.3995 | 0.2597 |
| *EVM0017087.1* | *Glyma.15G152000.1* | 0.2681 | 0.9671 | 0.2773 |
| *EVM0019301.1* | *Glyma.06G068800.1* | 0.1370 | 0.5681 | 0.2411 |
| *EVM0031497.1* | *Glyma.04G201900.1* | 0.2183 | 0.7362 | 0.2966 |
| *EVM0016298.1* | *Glyma.05G015900.1* | 0.0869 | 0.2886 | 0.3010 |
| *EVM0029153.1* | *Glyma.10G036600.1* | 0.1302 | 0.7367 | 0.1767 |
| *EVM0017798.1* | *Glyma.20G155200.1* | 0.1956 | 1.3312 | 0.1469 |
| *EVM0023826.1* | *Glyma.14G056200.1* | 0.1505 | 0.6272 | 0.2400 |
| *EVM0031318.1* | *Glyma.07G031200.1* | 0.1209 | 0.5765 | 0.2098 |
| *EVM0017483.1* | *Glyma.20G203500.1* | 0.1057 | 0.4295 | 0.2461 |
| *EVM0034637.1* | *Glyma.01G188600.1* | 0.1687 | 0.9752 | 0.1730 |
| *EVM0013260.1* | *Glyma.07G250100.1* | 0.1431 | 0.4728 | 0.3027 |
| *EVM0021979.1* | *Glyma.05G063500.1* | 0.1722 | 0.6810 | 0.2528 |
| *EVM0032511.1* | *Glyma.10G036700.1* | 0.1717 | 0.6552 | 0.2621 |
| *EVM0027510.1* | *Glyma.05G063600.1* | 0.1504 | 1.1206 | 0.1342 |
| *EVM0006777.1* | *Glyma.20G197000.1* | 0.1446 | 0.3555 | 0.4068 |
| *EVM0015149.1* | *Glyma.04G251400.1* | 0.1176 | 0.4073 | 0.2887 |
| *EVM0003995.1* | *Glyma.07G110000.1* | 0.1794 | 0.4852 | 0.3698 |
| *EVM0013392.1* | *Glyma.01G147600.1* | 0.2108 | 2.1623 | 0.0975 |
| *EVM0032787.1* | *Glyma.15G018400.1* | 0.1884 | 1.0012 | 0.1882 |
| *EVM0016285.1* | *Glyma.11G014800.1* | 0.2903 | 1.2403 | 0.2341 |
| *EVM0024520.1* | *Glyma.02G016100.1* | 0.1378 | 0.8105 | 0.1700 |
| *EVM0002293.1* | *Glyma.09G240400.1* | 0.1323 | 0.4324 | 0.3059 |
| *EVM0026285.1* | *Glyma.02G080200.1* | 0.1864 | 0.5861 | 0.3180 |
| *EVM0019801.1* | *Glyma.06G042100.1* | 0.1006 | 0.9467 | 0.1063 |
| *EVM0003729.1* | *Glyma.19G113100.1* | 0.1041 | 0.3403 | 0.3058 |
| *EVM0036388.1* | *Glyma.01G087500.1* | 0.1224 | 1.0645 | 0.1150 |
| *EVM0028339.1* | *Glyma.18G159900.1* | 0.0703 | 0.2996 | 0.2347 |
| *EVM0023141.1* | *Glyma.07G055000.1* | 0.1668 | 4.6133 | 0.0362 |
| *EVM0037078.1* | *Glyma.18G252400.1* | 0.2489 | 0.8447 | 0.2946 |
| *EVM0035983.1* | *Glyma.17G170300.1* | 0.0633 | 0.4844 | 0.1308 |
| *EVM0035087.1* | *Glyma.16G147500.1* | 0.3759 | 1.1303 | 0.3326 |
| *EVM0021339.1* | *Glyma.13G096900.1* | 0.0598 | 0.4332 | 0.1381 |
| *EVM0000779.1* | *Glyma.12G182400.1* | 0.2746 | 0.8317 | 0.3302 |
| *EVM0015734.1* | *Glyma.03G112100.1* | 0.3760 | 0.9567 | 0.3930 |
| *EVM0010624.1* | *Glyma.07G114300.1* | 0.3171 | 1.4148 | 0.2241 |
| *EVM0015677.1* | *Glyma.15G008600.1* | 0.0925 | 0.3002 | 0.3082 |
| *EVM0021893.1* | *Glyma.09G248200.1* | 0.1500 | 0.6349 | 0.2363 |
| *EVM0010310.1* | *Glyma.20G215700.1* | 0.2861 | 1.2049 | 0.2375 |
| *EVM0018957.1* | *Glyma.08G278800.1* | 0.5315 | 1.7076 | 0.3113 |
| *EVM0005801.1* | *Glyma.02G087400.1* | 0.2099 | 0.5455 | 0.3849 |
| *EVM0019855.1* | *Glyma.08G320700.1* | 0.1504 | 0.8867 | 0.1697 |
| *EVM0021771.1* | *Glyma.13G122600.1* | 0.1720 | 0.5118 | 0.3361 |
| *EVM0017315.1* | *Glyma.17G047300.1* | 0.1937 | 0.912 | 0.2124 |
| *EVM0032149.1* | *Glyma.03G162700.1* | 0.1897 | 0.779 | 0.2435 |
| *EVM0011666.1* | *Glyma.05G092800.1* | 0.1830 | 1.4888 | 0.1229 |
| *EVM0013242.1* | *Glyma.15G221600.1* | 0.1136 | 0.4586 | 0.2477 |
| *EVM0014070.1* | *Glyma.07G038200.1* | 0.0715 | 0.3419 | 0.2092 |
| *EVM0033695.1* | *Glyma.01G022500.1* | 0.1068 | 0.6979 | 0.1531 |
| *EVM0019590.1* | *Glyma.06G064000.1* | 0.0467 | 0.4737 | 0.0985 |
| *EVM0032083.1* | *Glyma.13G236600.1* | 0.0846 | 1.0916 | 0.0775 |
| *EVM0013295.1* | *Glyma.06G225200.1* | 0.0785 | 0.4221 | 0.1860 |
| *EVM0014312.1* | *Glyma.07G044300.1* | 0.1635 | 0.6040 | 0.2707 |
| *EVM0023142.1* | *Glyma.06G295300.1* | 0.1901 | 0.6132 | 0.3100 |
| *EVM0029311.1* | *Glyma.19G104200.1* | 0.2319 | 0.9781 | 0.2371 |
| *EVM0021580.2* | *Glyma.05G108600.1* | 0.0775 | 0.4238 | 0.1828 |
| *EVM0018988.1* | *Glyma.03G136100.1* | 0.0852 | 0.5158 | 0.1652 |
| *EVM0037148.1* | *Glyma.06G049200.1* | 0.1038 | 0.6230 | 0.1666 |
| *EVM0023336.1* | *Glyma.10G239400.1* | 0.1212 | 1.1730 | 0.1034 |
| *EVM0009457.1* | *Glyma.18G125200.1* | 0.0698 | 0.2677 | 0.2608 |
| *EVM0032499.1* | *Glyma.19G178200.1* | 0.1268 | 0.4543 | 0.2792 |
| *EVM0024005.1* | *Glyma.08G020900.1* | 0.2328 | 0.8430 | 0.2761 |
| *EVM0020293.1* | *Glyma.17G143900.1* | 0.2214 | 0.8200 | 0.2700 |
| *EVM0024679.1* | *Glyma.18G206600.1* | 0.1626 | 0.4829 | 0.3367 |
| *EVM0016025.1* | *Glyma.15G085400.1* | 0.1229 | 0.3752 | 0.3276 |
| *EVM0034544.1* | *Glyma.14G089200.1* | 0.1235 | 0.4176 | 0.2957 |

**Table S6.** Member numbers of each subfamily and group in the AP2/ERF family in *A. nanus* and other legumes species.

| Quantity of the subfamily and group member | DREB | | | | | | | ERF | | | | | | | | AP2 | | RAV | Soloist | Total |
| --- | --- | --- | --- | --- | --- | --- | --- | --- | --- | --- | --- | --- | --- | --- | --- | --- | --- | --- | --- | --- |
|  | A1 | A2 | A3 | A4 | A5 | A6 | Total | B1 | B2 | B3 | B4 | B5 | B6 | Total |  | |  | |  |  |
| *Glycine max* | 3 | 6 | 1 | 11 | 7 | 9 | 37 | 12 | 10 | 21 | 6 | 5 | 7 | 61 | 26 | | 2 | | 0 | 126 |
| *Medicago truncatula* | 4 | 7 | 1 | 14 | 11 | 4 | 41 | 17 | 6 | 16 | 6 | 5 | 16 | 66 | 21 | | 3 | | 0 | 131 |
| *Lotus japonicus* | 7 | 4 | 1 | 18 | 11 | 7 | 48 | 12 | 4 | 26 | 7 | 6 | 19 | 74 | 14 | | 2 | | 2 | 140 |
| *Cicer arietinum* | 6 | 5 | 1 | 14 | 10 | 7 | 43 | 12 | 5 | 23 | 14 | 8 | 14 | 76 | 24 | | 2 | | 2 | 147 |
| ***Ammopiptanthus nanus*** | **8** | **8** | **2** | **18** | **9** | **10** | **55** | **28** | **7** | **29** | **8** | **8** | **12** | **92** | **24** | | **2** | | **1** | **174** |
| *Cajanus cajan* | 5 | 9 | 1 | 18 | 10 | 7 | 50 | 16 | 5 | 39 | 8 | 7 | 23 | 98 | 25 | | 2 | | 1 | 176 |
| *Phaseolus vulgaris* | 8 | 8 | 1 | 19 | 10 | 8 | 54 | 17 | 4 | 33 | 9 | 8 | 24 | 95 | 26 | | 3 | | 1 | 179 |

**Table S7**. The primers used in qRT-PCR analysis

| Gene ID | Forward primer (5'-3') | Reverse primer (5'-3') |
| --- | --- | --- |
| EVM0000779.1 | CTCGAGGGTGATGATCTCGT | TCAACGCTGTCTTCATGGAC |
| EVM0026860.1 | ATGCTGACTTGGGCGTTTAT | GTCGTCAACCTTCTCCGTTT |
| EVM0007910.1 | TGATGAGGCTTTCAAACCAA | ACAAGATTTCCGTTCCACCA |
| EVM0005936.1 | ATGGAAAGGCCCTCCATATT | TCCAACCCAGTTGAATCCTC |
| EVM0023336.1 | GAGTCGGAGACGGATGAAAG | CTCCATGTTCCTCAGCCACT |
| EVM0023826.1 | TGCAGCTGAAGATGTGAAGG | TGGGGAGGCAGATTCATAAG |
| EVM0005020.1 | CCTCAAACGGATCACCAAGT | TGAACCACCTTTGGTTCCTC |
| EVM0012736.1 | CGCCTATCAAAACTGGGAAG | ATTGACTTGCCACCCCTTTT |
| EVM0024679.1 | GGGCCTTTCAAGAATGTCAA | AGGCTCCTTCTGCATGTGAT |
| EVM0009472.1 | CTCTTGCAAACGCAGTCAGT | CACAACACCCCCTTATTGCT |
| EVM0023141.1 | GGCTCACATGATGATGAAGG | GGAAAAAGTCCACCACTTGC |
| EVM0032787.1 | GGGGTACGAGGTAACGGAAT | GGTTTGGATTGGAAGTTGGA |
| EVM0013392.1 | TGATGGGTGGCCCTATACTC | GCGGGAGCAAATGCTTAATA |
| EVM0020293.1 | TCGAGCCACACACAAGAATC | AGCCAAGCCGATGAGTAATG |
| EVM0026889.1 | CGGAGGAGTTGGGTGAGATA | TGCTGTGATACCAAGGGTGA |
| EVM0027012.1 | TCATTGTGTTCTGAAGGGTCA | GACCCCATTGGCTGATACAC |
| EVM0003995.1 | CCGGCTAGAGGAGCAATTTT | TTGTGCTATCAGAGTGCAGTGA |
| EVM0016549.1 | CCCGCTCTCCTGATCATAAA | CGTTCCATGTCAATTCTCCA |
| EVM0035043.1 | CAGTTTGCTGGATGACATGG | CAAACCTCACACATGGATGC |
| EVM0026054.1 | ATGGAAACCCTCCTCCTTGT | CGAGGACTTGGAGTTGATGG |
| EVM0017315.1 | TTCAGAGTTGCAGCATACGG | TCCGGAAAAGAACCATTGAG |
| EVM0019801.1 | GGCCTCGTCACAGTACTCCT | CTTCGGGTTTTGGATACTCG |
| EVM0012385.1 | TCCGATTTTGGCCTCTATTC | AAGCCTGCAGGTGAATCAAA |
| EVM0014092.1 | TCCAGCAAGGGGATATGTCT | GGAGTTCGAAGAAGCATGGA |
| EVM0034637.1 | AGATACCCGAACCCGAGAAT | CAAAGGCCTCATCAACAACA |
| EVM0014569.1 | TGACTCGGTGTCACCCTTC | TCATCGGTGGTCTCTCTGTG |
| EVM0034483.1 | TGCAAAGTTGGAGTCTGACG | TCCAAATCCAATCCGAAGTT |
| EVM0017791.1 | CATGAGGCCTTTCTTCTGGA | GGGGTGGAAGTAATCAGCAA |
| EVM0016285.1 | GGCCTTCTTCATTGGACAAA | TCAACATCCATGGCAACATC |
| EVM0032643.1 | TTCGACAGAGTGTCCCATGA | CCAATTACCCACCAAGACCA |
| EVM0006606.1 | GAATCGTTTCTACGGCAGGA | GGACGAAAGTGTTCTCCTTGTT |
| EVM0023142.1 | ATTCCCACACCAATTTGGAA | ATGTTGTGCTGTGACCCAAA |
| eIF1 | CTGACATGCGCCGTAGGAACG | CCCTGCTTATGCCAGTCTTTT |

**At**

**An**

**A1**

**At**

**An**

**A2**

**At**

**An**

**A3**

**At**

**An**

**A4**

**At**

**An**

**A5**

**At**

**An**

**A6**

**Figure S1.** Comparison of deduced amino acid sequences of the AP2-domain of the DREB subfamily proteins from *A. thaliana* (At) and *A. nanus* (An)*.* The black background represents conserved amino acid residues in each group.

**At**

**An**

**B1**

**At**

**An**

**B2**

**At**

**An**

**B3**

**At**

**An**

**B4**

**At**

**An**

**B5**

**At**

**An**

**B6**

**Figure S2.** Comparison of deduced amino acid sequences of the AP2 domain of the ERF subfamily proteins from *A. thaliana* (At) and *A. nanus* (An)*.*

**At**

**An**

**RAV**

**AP2/ERF domain**

**B3 domain**

**Figure S3.** Comparison of deduced amino acid sequences of the AP2 domain and B3 domain of the RAV subfamily proteins from *A. thaliana* (At) and *A. nanus* (An)*.*

**At**

**An**

**AP2**

**AP2/ERF domain I**

**AP2/ERF domain II**

**Figure S4.** Comparison of deduced amino acid sequences of the AP2-domain of the AP2 subfamily proteins from *A. thaliana* (At) and *A. nanus* (An)*.*


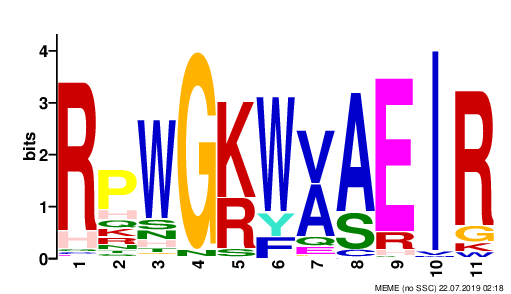

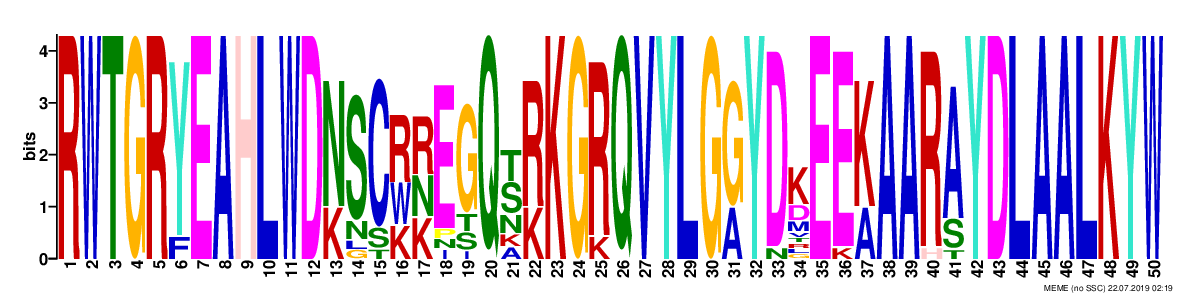

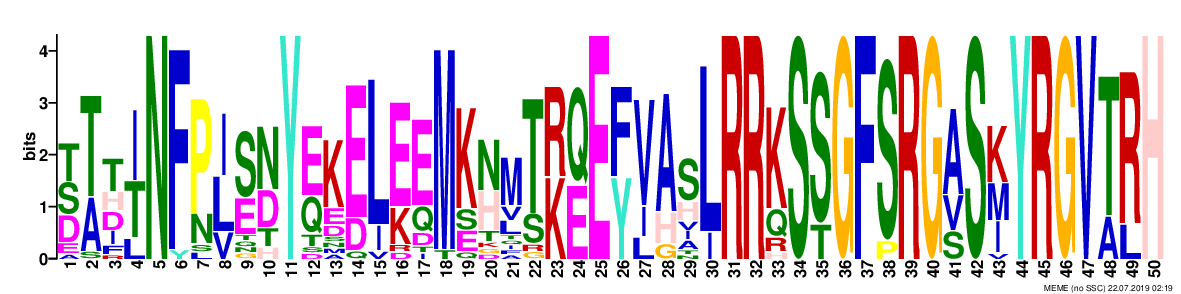

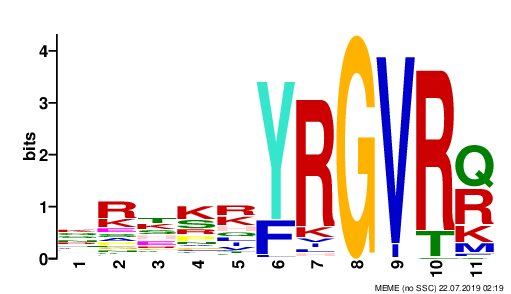

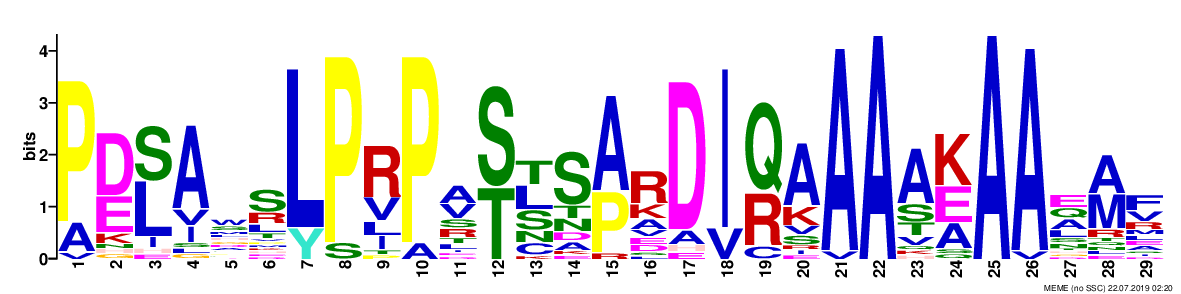

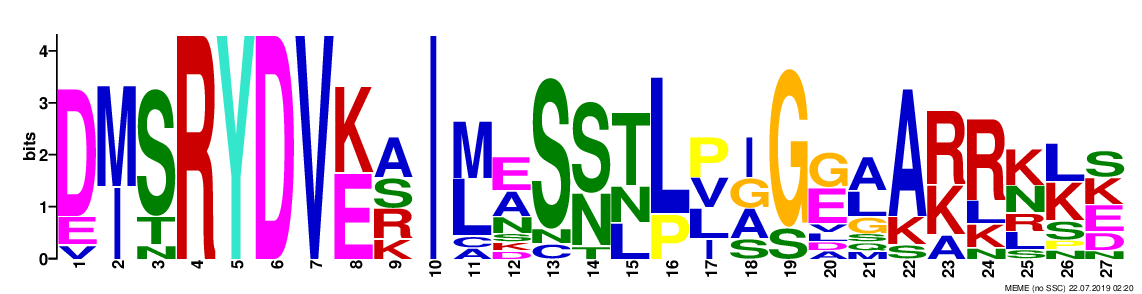

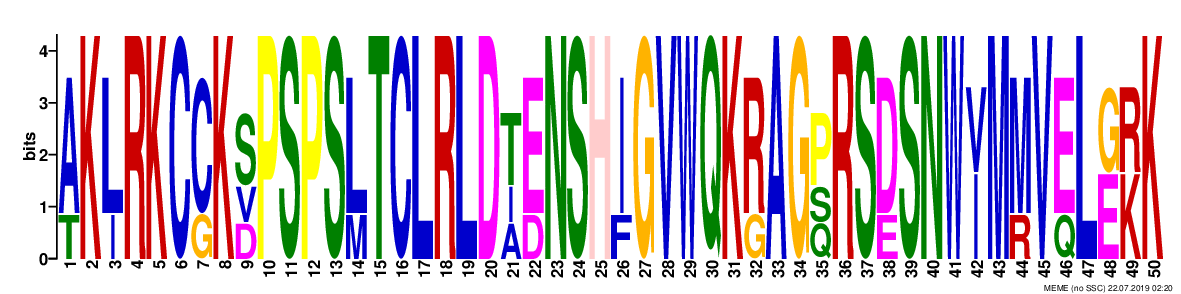

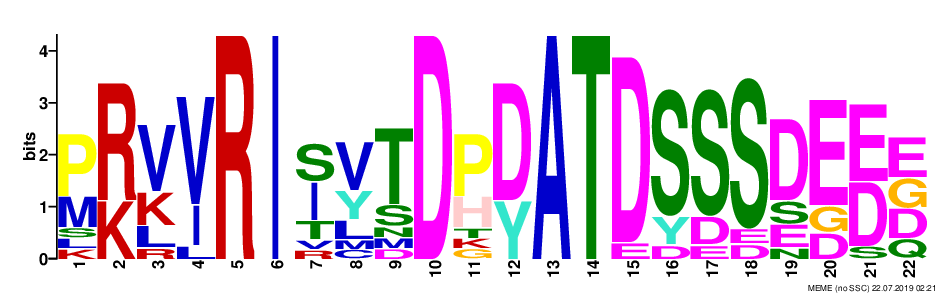

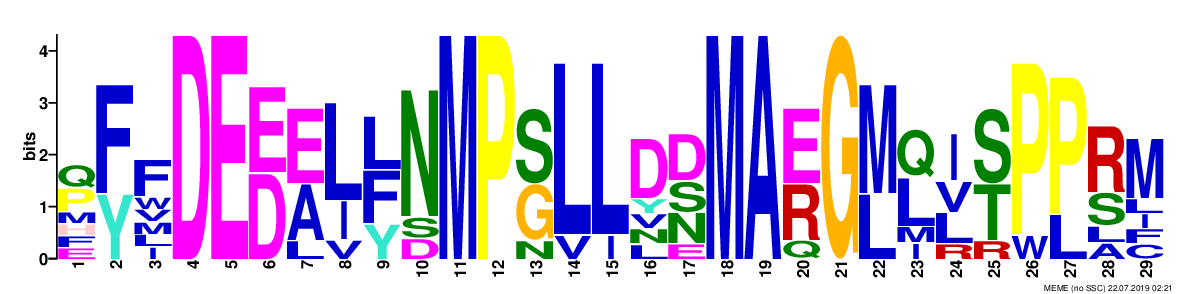

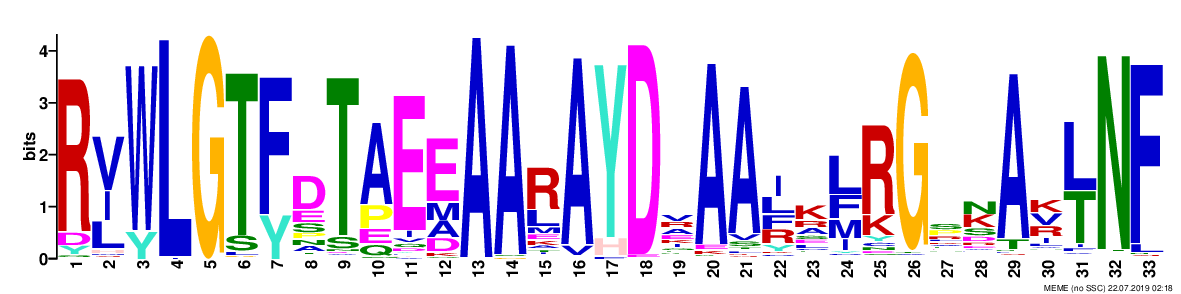


**Motif 1**

**Motif 8**

**Motif 7**

**Motif 6**

**Motif 5**

**Motif 4**

**Motif 3**

**Motif 2**

**Motif 10**

**Motif 9**


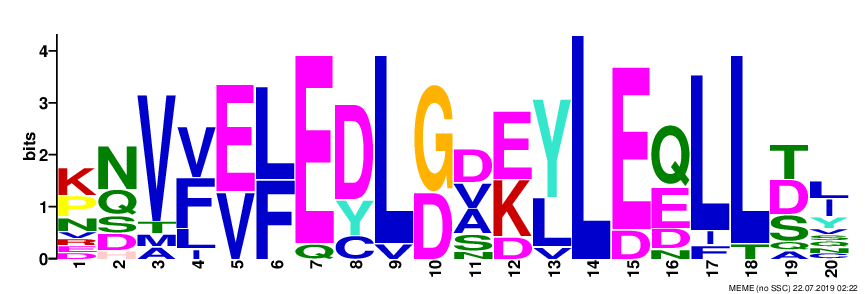

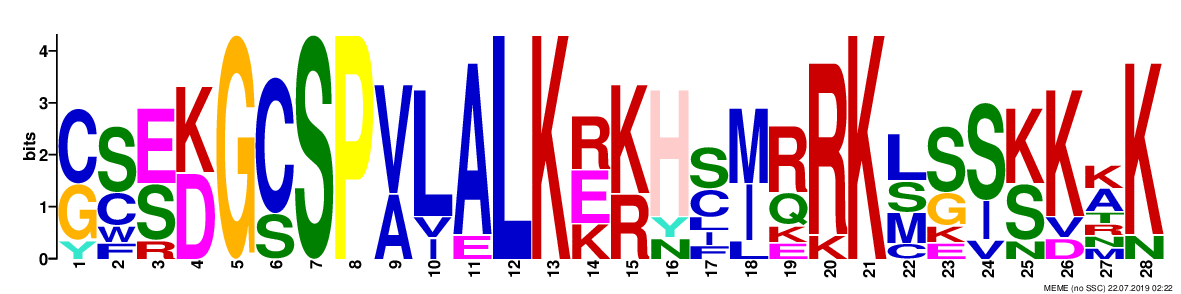

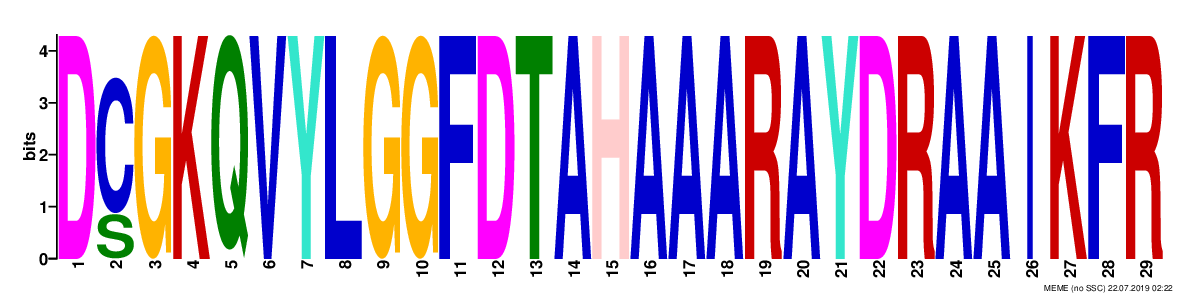

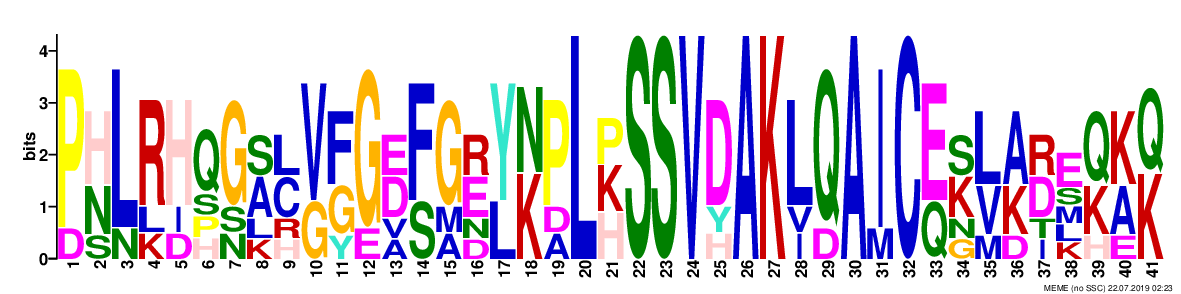

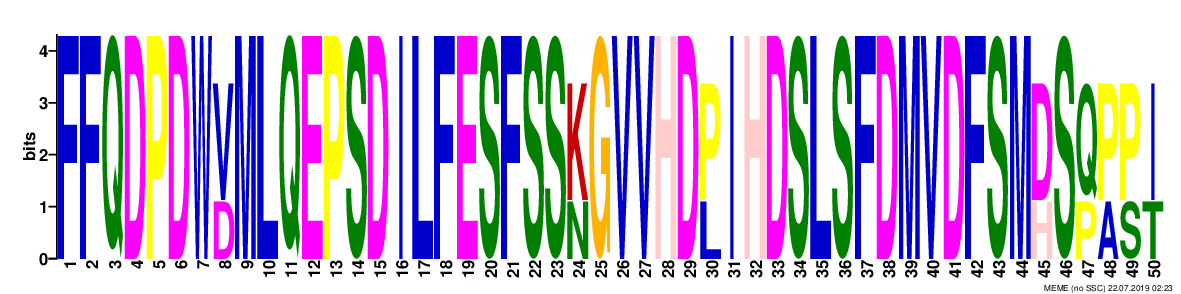

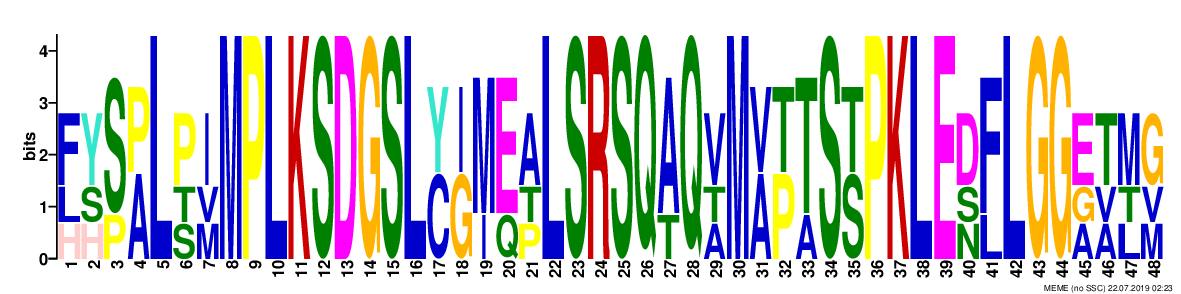

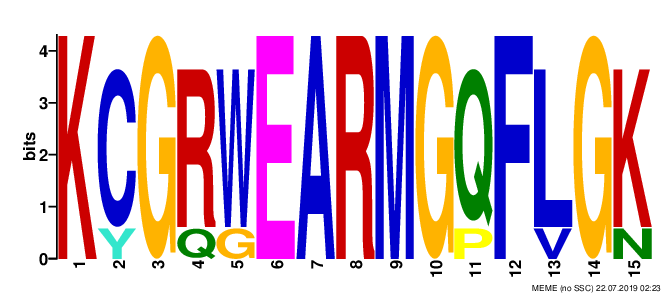

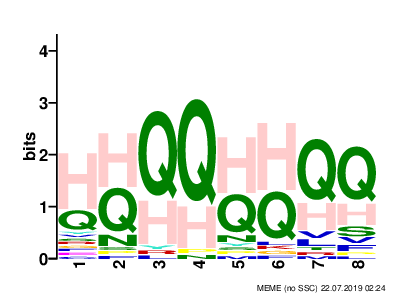

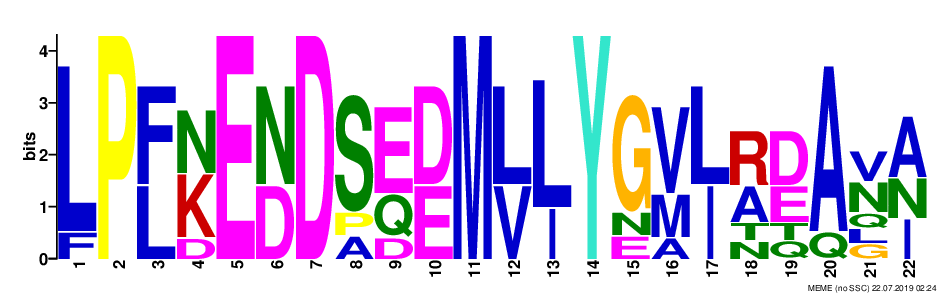

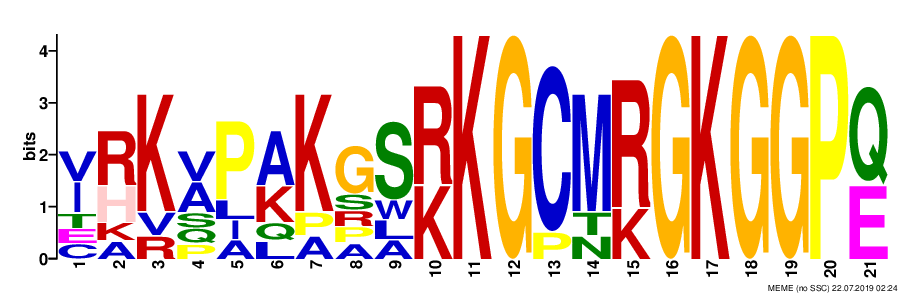


**Motif 11**

**Motif 18**

**Motif 17**

**Motif 16**

**Motif 15**

**Motif 14**

**Motif 13**

**Motif 12**

**Motif 20**

**Motif 19**

**Figure S5.** The twenty conserved motifs identified in the AP2/ERF family members





**Figure S6.** Motif compositions and gene structures of the AP2/ERF family members in *A. nanus*. (A) Conserved motifs analysis of the AnAP2/ERF family transcription factors. All motifs were identified using the MEME database. (B) Gene structure of AnAP2/ERF family members. Exons and introns are represented by yellow boxes and black lines, respectively, the UTR (Un-Translated Region) is shown in green. To make the figure clear and readable, the intron-exon graphs of two genes with very long introns (*EVM0005020.1* and *EVM0027377.1*) were not drawn in scale.
